# Resveratrol-induced Sirt1 phosphorylation by LKB1 mediates mitochondrial metabolism

**DOI:** 10.1016/j.jbc.2021.100929

**Published:** 2021-07-01

**Authors:** Yuanyuan Huang, Jianlin Lu, Li Zhan, Ming Wang, Ronghua Shi, Xiao Yuan, Xinjiao Gao, Xing Liu, Jianye Zang, Wei Liu, Xuebiao Yao

**Affiliations:** 1 MOE Key Laboratory for Cellular Dynamics, University of Science & Technology of China School of Life Sciences, Hefei, China; 2 Anhui Key Laboratory for Cellular Dynamics & Chemical Biology, CAS Center for Excellence in Molecular Cell Science & Hefei National Science Center for Physical Sciences at Microscale, Hefei, Anhui, China; 3 Keck Center for Organoids Plasticity, Morehouse School of Medicine, Atlanta, Georgia, USA; 4 Department of Biochemistry and Department of Cardiology of the Second Affiliated Hospital, Zhejiang University School of Medicine, Hangzhou, Zhejiang, China

**Keywords:** mitochondria, phosphorylation, LKB1, Sirt1, deacetylation, DBC1, deleted in breast cancer 1, ESA, essential for Sirt1 activity, FA, Formic acid, LKB1, liver kinase B1, LZ, leucine zipper, mtDNA, mitochondrial DNA

## Abstract

The NAD^+^-dependent deacetylase Sirt1 has been implicated in the prevention of many age-related diseases, including cancer, type 2 diabetes, and cardiovascular disease. Resveratrol, a plant polyphenol, exhibits antiaging, antitumor, and vascular protection effects by activating Sirt1. However, the molecular mechanism of Sirt1 activation as induced by resveratrol remains unclear. By knockdown/rescue experiments, fluorometric Sirt1 activity assay, immunoprecipitation, and pull-down assays, we identify here that the tumor suppressor LKB1 (liver kinase B1) as a direct activator of Sirt1 elicited by resveratrol. Resveratrol promotes the binding between LKB1 and Sirt1, which we first reported, and this binding leads to LKB1-mediated phosphorylation of Sirt1 at three different serine residues in the C terminus of Sirt1. Mechanistically, LKB1-mediated phosphorylation increases intramolecular interactions in Sirt1, such as the binding of the C terminus to the deacetylase core domain, thereby eliminating DBC1 (Deleted in Breast Cancer 1, Sirt1 endogenous inhibitor) inhibition and promoting Sirt1–substrate interaction. Functionally, LKB1-dependent Sirt1 activation increases mitochondrial biogenesis and respiration through deacetylation and activation of the transcriptional coactivator PGC-1α. These results identify Sirt1 as a context-dependent target of LKB1 and suggest that a resveratrol-stimulated LKB1-Sirt1 pathway plays a vital role in mitochondrial metabolism, a key physiological process that contributes to numerous age-related diseases.

Protein deacetylase Sirt1 is a member of the Sirtuin family that utilizes NAD^+^ as a cofactor (1, 2). It plays important roles in numerous fundamental cellular processes, including aging, energy metabolism, differentiation, and genomic stability (3, 4). Cellular functions of Sirt1 are modulated through deacetylation of targets such as p300 (5), p53 (6), Forkhead box (FOXO) (7, 8), peroxisome proliferator-activated receptor γ (PPARγ) (9), and PPARγ coactivator PGC-1α (10). It has long been postulated that the activity of Sirt1 is modulated in response to multiple stresses, including genotoxicity, energetic stress, and oxidative stress (11). Therefore, it is of great significance to understand the activation mechanisms of Sirt1 in these distinct contexts.

Mounting evidence demonstrates that Sirt1 activity is regulated in three different ways. First, as an NAD^+^-dependent deacetylase, Sirt1 activity is coupled to dynamic NAD^+^/NADH ratio in response to cellular metabolic status (1, 2, 12). Second, Sirt1 activity is regulated by physical interaction with other proteins such as DBC1 (Deleted in Breast Cancer 1). DBC1 interacts with the catalytic domain of Sirt1 and inhibits Sirt1 deacetylase activity both *in vivo* and *in vitro* (13, 14). Recent study showed that insulin-mediated liver PACS-2 is another Sirt1 inhibitor and suppresses Sirt1 activity (15). Posttranslational modifications such as phosphorylation, sumoylation, and O-GlcNAcylation also regulate Sirt1 activity (16, 17, 18). In particular, recent evidence indicated that phosphorylation is an important regulatory mechanism underlying Sirt1 activity control (19, 20, 21, 22, 23).

The polyphenol resveratrol (2,3,4′-trihydroxystilbene) is an antiviral toxin secreted by plants in response to environmental stress and began to enter into public horizon when it is associated with cardiovascular benefits found in red wine (24). As a calorie restriction mimetic (25, 26, 27, 28, 29), resveratrol exhibits protection against aging and possesses health benefits in a Sirt1-dependent manner (26, 30). At the cellular level, it is generally believed that resveratrol regulates mitochondrial function (30, 31). Several studies have linked the mitochondrial homeostasis to PGC-1α and Sirt1 deacetylase (10, 32, 33, 34, 35). Recent effort in screening for small molecular activators of Sirt1 revealed the potent action of resveratrol (36). However, the molecular mechanisms underlying resveratrol-regulated mitochondrial bioenergetics, metabolism, and signaling remain elusive.

Several kinases have been reported to phosphorylate Sirt1 and regulate its deacetylase activity (19, 20, 21, 22, 23). However, we found that depletion of any of these kinases did not affect Sirt1 phosphorylation triggered by resveratrol treatment. Since the association between liver kinase B1 (LKB1) and Sirt1 has been described previously (37), we predicted and then demonstrated that LKB1 directly affects Sirt1 activation. LKB1 (also known as STK11) formed a 1:1:1 heterotrimeric complex with the pseudokinase STRAD (STE20-related adaptor) and the scaffolding protein MO25 (mouse protein 25) in cells (38, 39). There are two isoforms of both STRAD (STRADα or STRADβ) and MO25 (MO25α or MO25β) that have similar interaction with LKB1. Unlike the majority of protein kinases, activated by upstream kinase phosphorylation, LKB1 is activated by binding to STRAD and MO25 in response to energy stress (40, 41, 42). Upon activation, LKB1 regulates downstream kinases such as AMPK and AMPK-related kinases to orchestrate diversified cellular functions including catabolism and cellular homeostasis (43, 44, 45). In addition, LKB1 regulates cellular polarity, metastasis, and mitosis (46, 47, 48). Mutations in LKB1 have been linked to Peutz-Jeghers syndrome (49). Loss of LKB1 leads to metabolic alterations that drive tumorigenesis (46, 50, 51, 52). Therefore, LKB1 was initially recognized as a tumor suppressor. However, recent studies have also shown LKB1 function as an oncogene that is crucial for cancer survival (53, 54). Thus, it was of great importance to delineate the context-dependent function of LKB1 in cell fate decision.

Here, we show that resveratrol treatment promotes LKB1 to interact with Sirt1 and subsequent phosphorylation of the C terminus of Sirt1. LKB1-mediated phosphorylation increases the binding capacity between the C terminus and deacetylase core of Sirt1, releases the DBC1-induced intermolecular inhibition, and thus promotes the deacetylase core–substrate interaction. Thus, Sirt1 is a novel context-dependent substrate of LKB1 and the LKB1-Sirt1 pathway orchestrates resveratrol-elicited mitochondrial function.

## Results

### LKB1-dependent phosphorylation is required for resveratrol-induced Sirt1 activation

**Figure 1:**
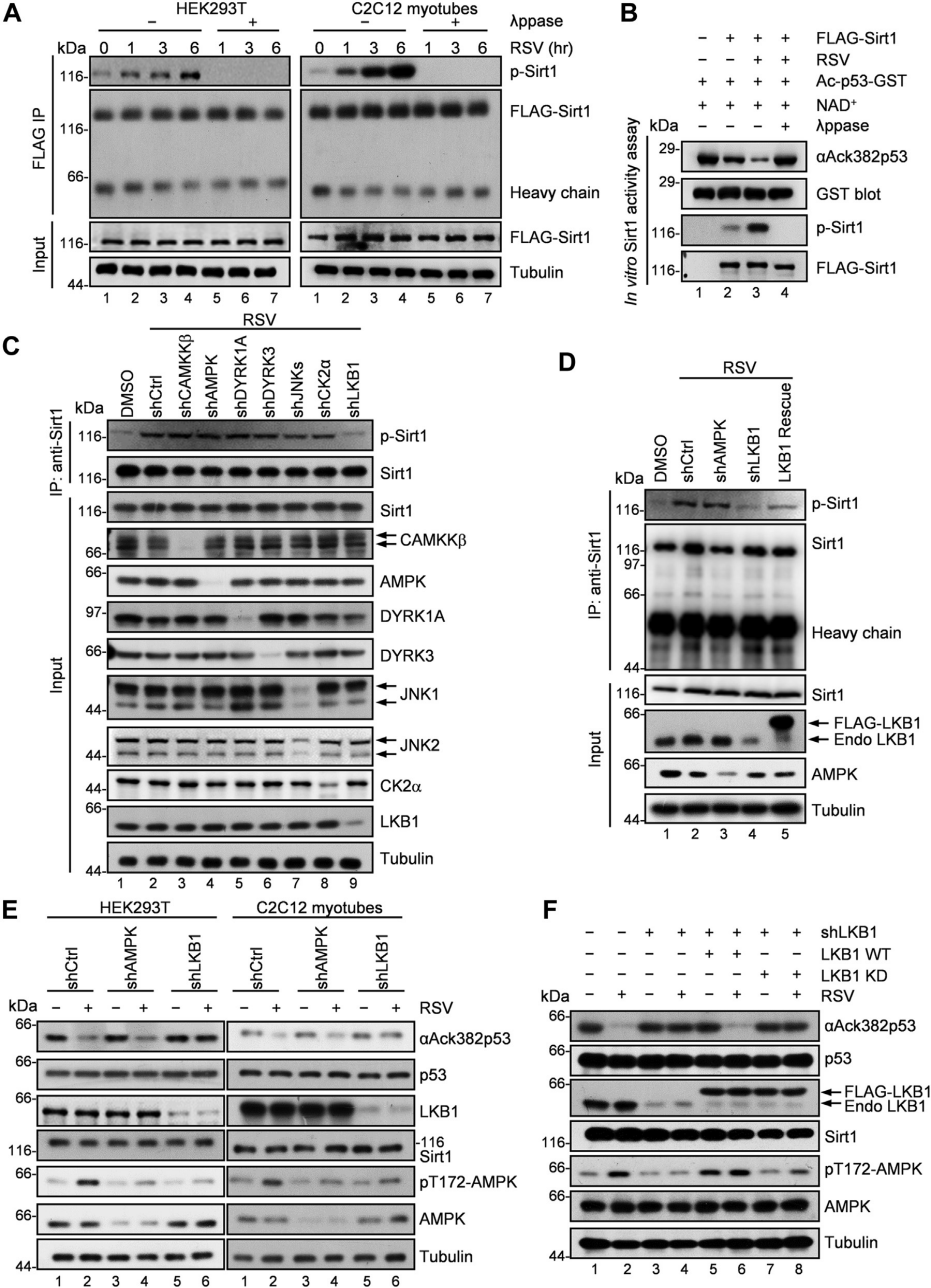
**LKB1-dependent phosphorylation is required for resveratrol-induced Sirt1 activation.***A*, phosphorylation of Sirt1 in cells treated with resveratrol. HEK293T cells were infected with lentivirus-based particles expressing FLAG-Sirt1 for 12 h, and 48 h later cells were treated with 25 μM resveratrol for 6 h. Cells were collected at indicated time points and FLAG-Sirt1 proteins were immunoprecipitated by anti-FLAG and immunoblotted with anti-phospho-serine/threonine. Immunoprecipitated FLAG-Sirt1 proteins treated with lambda protein phosphatase at 30 °C for 30 min were negative controls. For C2C12 myoblasts, cells were infected with virus particles for 12 h. After infection, cells were continued to culture with fresh medium for 48 h and then were grown in DMEM with 2% horse serum for 4 days to generate C2C12 myotubes. The next steps were same as the mentioned process for HEK293T cells. Representative of three independent experiments. *B*, *in vitro* Sirt1 deacetylase activity assay. HEK293T cells were infected with lentivirus-based particles expressing FLAG-Sirt1 for 12 h, and 48 h later cells were treated with 25 μM resveratrol for 6 h. FLAG-Sirt1 proteins were immunoprecipitated by anti-FLAG and eluted by 3×FLAG peptide. Then 100 ng eluted FLAG-Sirt1 was incubated with 1 mM NAD^+^ and 2 μg GST-tagged K382ac p53 peptide from *E. coli* at 37 °C for 30 min in 40 μl Sirt1 assay buffer. The acetylation level of K382 site was analyzed by using anti-acetyl-p53 K382 antibody. The precipitated FLAG-Sirt1 pretreated with lambda protein phosphatase and then subjected to *in vitro* deacetylation assay was the negative control. Representative of three independent experiments. p53 K382 is the deacetylation site of Sirt1 and ack382-p53 is the marker of Sirt1 activity (6, 86). *C*, phosphorylation of Sirt1 in gene-depleted HEK293T cells treated with resveratrol. HEK293T cells were infected with lentivirus-based particles expressing shRNA control, CAMKKβ shRNA, AMPK shRNA, DYRK1A shRNA, DYRK3 shRNA, JNKs (JNK1 and JNK2) shRNAs, CK2α shRNA, or LKB1 shRNA for 12 h, and 48 h later cells were treated with 25 μM resveratrol for 6 h. Sirt1 proteins were immunoprecipitated by anti-Sirt1 antibody and immunoblotted with anti-phospho-serine/threonine. Representative of three independent experiments. *D*, phosphorylation of Sirt1 in resveratrol-treated LKB1-depleted HEK293T cells expressing FLAG-tagged WT LKB1. HEK293T cells were infected with lentivirus-based particles expressing shRNA control, AMPK shRNA, or LKB1 shRNA for 12 h, and 36 h later LKB1-depleted cells were infected with virus particles expressing FLAG-LKB1 for 12 h. After 36 h, cells were treated with 25 μM resveratrol for 6 h. Sirt1 proteins were immunoprecipitated by anti-Sirt1 antibody and immunoblotted with anti-phospho-serine/threonine. Representative of three independent experiments. *E*, the deacetylase activity of Sirt1 in resveratrol-treated LKB1-depleted cells. HEK293T cells were infected with lentivirus-based particles expressing shRNA control, AMPK shRNA, or LKB1 shRNA for 12 h. After 48 h, cells were pretreated with 1 μM doxorubicin for 1 h to increase *in vivo* K382 acetylation of p53 (deacetylation site of Sirt1) and then were treated with 25 μM resveratrol for 6 h. The whole-cell lysate (WCL) was immunoblotted with anti-acetyl-p53 K382. For C2C12 myoblasts, cells were first were infected with virus particles for 12 h. After infection, cells were continued to culture in fresh DMEM for 48 h and then were grown in DMEM with 2% horse serum for 4 days to generate myotubes. The next steps are same as the mentioned process for HEK293T cells. Representative of three independent experiments. (44). The p53 K382 is the deacetylation site of Sirt1 and ack382-p53 is the marker of Sirt1 activity (6, 86). *F*, the deacetylase activity of Sirt1 in resveratrol-treated LKB1-depleted HEK293T cells expressing WT LKB1 or kinase-dead mutant. HEK293T cells were infected with lentivirus-based particles expressing shRNA control or LKB1 shRNA for 12 h, and 36 h later LKB1-depleted cells were infected with virus particles expressing FLAG-LKB1 (WT or the KD mutant) for 12 h. After 36 h, cells were treated with 25 μM resveratrol for 6 h. WCL were immunoblotted with anti-acetyl-p53 K382. Endo LKB1, endogenous LKB1; KD, kinase dead; LKB1, Lys78Met; NAD, nicotinamide adenine dinucleotide, Sirt1 cofactor; pT172-AMPK, marker of LKB1 activity; RSV, resveratrol; (44).

To delineate the potential mechanism underlying resveratrol-elicited Sirt1 activation, we first treated cells with resveratrol and measured Sirt1 activity by a fluorophore-conjugated acetylated p53 peptide (substrate of Sirt1) (55). As shown in Fig. S1*A*, resveratrol activated Sirt1 and exhibited the highest activity among the six drugs tested, including four natural polyphenols that stimulate Sirt1 activity (36) and two chemical inhibitors of Sirt1 that are shown as negative controls (EX527 and Nicotinamide). Of interest, treatment with these natural polyphenols led to serine/threonine phosphorylation of Sirt1 and resveratrol showed to be the most prominent (Fig. S1*B*). To confirm the effect of resveratrol treatment on Sirt1 phosphorylation, aliquots of 293T and C2C12 cells were infected with lentivirus-based particles expressing FLAG-Sirt1 followed by resveratrol treatment and then subjected to immunoprecipitation at different time points. The isolated FLAG-Sirt1 proteins were probed for pan-phosphorylated Ser/Thr. As shown in Figure 1*A*, treatment of cells with resveratrol time-dependently increased the phosphorylation levels of Sirt1, suggesting that phosphorylation of Sirt1 is a functional readout of enzymatic activity.

To further determine that the increased Sirt1 phosphorylation is coupled to Sirt1 activation, we performed *in vitro* Sirt1 activity assay by incubating FLAG-Sirt1 isolated from 293T cells with recombinant acetylated p53-GST (p53-K382ac) purified from *Escherichia coli* using a recently reported genetically encoded method with unnatural amino acids (56) (Fig. S1*C*). As shown in Figure 1*B*, in the presence of NAD^+^, Ac-p53-GST was deacetylated by phosphorylated FLAG-Sirt1 from resveratrol-treated cells, but not by unphosphorylated FLAG-Sirt1 from cells treated with resveratrol and phosphatase (lane 4), suggesting that Sirt1 is activated by the phosphorylation (Fig. S1*D*).

Several kinases have been reported to phosphorylate Sirt1 (19, 20, 21, 22, 23). However, depletion any of these kinases did not affect Sirt1 phosphorylation triggered by resveratrol. To probe for the kinase responsible for Sirt1 phosphorylation elicited by resveratrol treatment, we carried out siRNA-mediated knockdown. As shown in Figure 1*C* and Fig. S1*E*, Sirt1 phosphorylation was dramatically reduced in LKB1-depleted cells, suggesting that LKB1 is the potential upstream kinase for Sirt1 phosphorylation. Of importance, upon reconstitution of wildtype but not a kinase-dead LKB1 in the knockdown cells, both the phosphorylation level and the deacetylase activity of Sirt1 were restored (Fig. 1, *D*–*F*, Fig. S1, *F* and *G*). To reconfirm resveratrol-elicited Sirt1 phosphorylation is medicated by LKB1, we employed a structurally distinct Sirt1 activator SRT1720 (57). As shown in Fig. S1*H*, Sirt1 activity is elicited by the chemical activator of Sirt1 and activation of Sirt1 requires LKB1 kinase activity (lanes 4 and 6). Previous studies have reported that AMPK mediates Sirt1 activation by increasing cellular NAD^+^ or GAPDH-Sirt1 interaction (58, 59); we therefore measured the deacetylase activity of Sirt1 in AMPK knockdown cells. Our results suggested that AMPK is not required for Sirt1 phosphorylation and activation by resveratrol (Fig. 1, *D* and *E*, and Fig. S1*I*). Thus, we conclude that LKB1 kinase activity is responsible for Sirt1 phosphorylation and activation by resveratrol.

### LKB1 activates Sirt1 by direct phosphorylation

**Figure 2:**
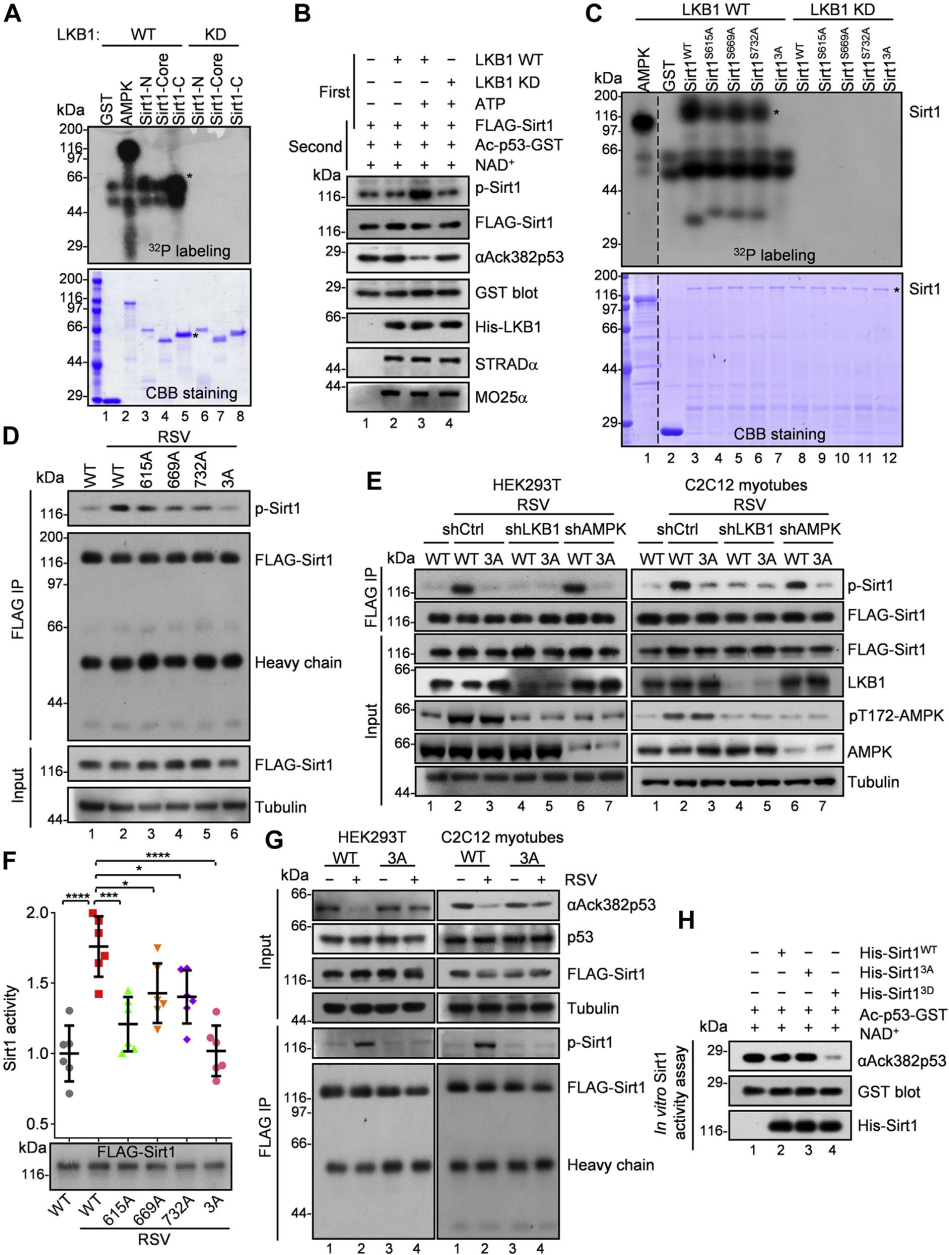
**LKB1 activates Sirt1 by direct phosphorylation.***A*, *in vitro* kinase assay using recombinant LKB1 kinase and purified GST-Sirt1 truncations. Samples were immunoblotted as indicated. MBP-AMPK incubated with LKB1 kinase was as positive control. *Asterisks* mark the C terminus of Sirt1. *B*, LKB1-dependent phosphorylation of Sirt1 is positively correlated with its deacetylase activity. HEK293T cells were infected with lentivirus-based particles expressing FLAG-Sirt1 and FLAG-Sirt1 was immunopurified by anti-FLAG. Precipitated FLAG-Sirt1 (2 μg), 100 ng recombinant LKB1 kinase (WT or kinase dead mutant), and 50 μM ATP were incubated in 40 μl kinase assay buffer at 30 °C for 30 min. FLAG-Sirt1 was spun down and 100 ng FLAG-Sirt1 was incubated with 1 mM NAD^+^ and 2 μg GST-tagged K382ac p53 peptide at 37 °C for 30 min in 40 μl Sirt1 assay buffer. The acetylation level of K382 site was analyzed by using anti-acetyl-p53 K382 antibody, and the phosphorylation level of Sirt1 was analyzed by using anti-phospho-serine/threonine. Representative of three independent experiments. p53 K382 is the deacetylation site of Sirt1 and ack382-p53 is the marker of Sirt1 activity (6, 86). *C*, *in vitro* kinase assay using recombinant LKB1 kinase and purified GST-Sirt1 WT or mutants. Samples were subjected to immunoblotting as indicated. *Asterisks* mark the proteins of interest. *D*, HEK293T cells stably expressing Sirt1 shRNA were transfected with lentivirus-based particles expressing FLAG-Sirt1 WT, FLAG-Sirt1 S615A, FLAG-Sirt1 S669A, FLAG-Sirt1 S732A, or FLAG-Sirt1 3A for 12 h. After 48 h, cells were treated with 25 μM resveratrol for 6 h. FLAG-Sirt1 WT or mutant proteins were immunoprecipitated by anti-FLAG. Phosphorylation level of Sirt1 was analyzed by using anti-phospho-serine/threonine. The 3A mutation, Ser615, Ser669, and Ser732 were replaced by Ala. *E*, phosphorylation of Sirt1 in resveratrol-treated LKB1-depleted cells expressing FLAG-Sirt1 WT or 3A mutant. Cells were infected with lentivirus-based particles expressing Sirt1 shRNA, and particles expressing shRNA control, AMPK shRNA, or LKB1 shRNA for 12 h, and 36 h later gene-depleted cells were infected with virus particles expressing FLAG-Sirt1 WT or 3A mutant for 12 h. After 36 h, cells were treated with 25 μM resveratrol for 6 h. FLAG-Sirt1 proteins were immunoprecipitated and immunoblotted with anti-phospho-serine/threonine. Representative of three independent experiments. The pT172-AMPK, marker of LKB1 activity. *F*, the activity of Sirt1 WT or mutants that were quantified by using a fluorophore-conjugated acetylated p53 peptide. HEK293T cells were lentivirus-based particles expressing Sirt1 shRNA for 12 h, and 36 h later gene-depleted cells were infected with virus particles expressing FLAG-Sirt1 WT or mutants for 12 h. After 48 h, cells were treated with 25 μM resveratrol for 6 h. FLAG-Sirt1 proteins were immunopurified by anti-FLAG and eluted by 3×FLAG peptide. And 50 ng eluted FLAG-Sirt1 were incubated with 1 mM of NAD^+^ and 200 μM fluorescently labeled acetylated p53 peptide in Sirt1 assay buffer at 37 °C for 30 min and the reaction was stopped with developer solution containing 2 mM nicotinamide. Sirt1 activity was assessed by measuring the fluorescent emission at 460 nm, following excitation at 360 nm. Data represents mean ± SD. The experiment was repeated six times independently. Statistical significance was determined by Dunnett's multiple comparisons test. ∗∗∗∗*p* (WT, WT RSV) < 0.0001. ∗∗∗*p* (WT, 615A) = 0.0002. ∗*p* (WT, 669A) = 0.0283. ∗*p* (WT, 732A) = 0.0164. ∗∗∗∗*p* (WT, 3A) < 0.0001. 615A, 669A, 732A, and 3A were replaced by Ala. *G*, LKB1-mediated phosphorylation of Sirt1 is positively correlated with its deacetylase activity in resveratrol-treated Sirt1-depleted cells expressing FLAG-Sirt1 WT or 3A mutant. Cells were infected with lentivirus-based particles expressing Sirt1 shRNA for 12 h, and 36 h later gene-depleted cells were infected with virus particles expressing FLAG-Sirt1 WT or 3A mutant for 12 h. After 36 h, cells were pretreated with 1 μM doxorubicin for 1 h to increase *in vivo* K382 acetylation of p53 (deacetylation site of Sirt1) and then were treated with 25 μM resveratrol for 6 h. The whole-cell lysate (WCL) was immunoblotted with anti-acetyl-p53 K382. FLAG-Sirt1 proteins were immunoprecipitated and immunoblotted with anti-phospho-serine/threonine. Representative of three independent experiments. The p53 K382 is the deacetylation site of Sirt1 and ack382-p53 is the marker of Sirt1 activity (6, 86). *H*, *in vitro* Sirt1 deacetylase activity assay. Baculovirus expressed His-tagged Sirt1 deacetylase (aa 193–747, Abcam, recombinant human Sirt1 protein, ab101130) WT, 3A mutant, or 3D mutant was incubated with 1 mM NAD^+^ and 2 μg GST-tagged K382ac p53 peptide from *Escherichia coli* at 37 °C for 30 min in 40 μl Sirt1 assay buffer. Acetylation level of K382 site was analyzed by using anti-acetyl-p53 K382 antibody. Representative of three independent experiments. The 3D mutation, Ser615, Ser669, and Ser732 were replaced by Asp. The p53 K382 is the deacetylation site of Sirt1 and ack382-p53 is the marker of Sirt1 activity (6, 86). 3A, Ser615, Ser669, and Ser732; 615A, Ser615Ala; 669A, Ser669Ala; 732A, Ser732Ala; KD, kinase dead; LKB1, Lys78Met; RSV, resveratrol; Sirt1-C, C terminus, aa 511 to 747; Sirt1-Core, core domain, aa 234 to 510; Sirt1-N, N terminus, aa 1 to 233.

Because LKB1 is required for resveratrol-induced Sirt1 phosphorylation, we asked whether Sirt1 could be a novel substrate of LKB1. We first performed an *in vitro* kinase assay using recombinant LKB1-STRADα-Mo25α complex (42) by autoradiography with ^32^P-labeled ATP and confirmed the phosphorylation at the C terminus of Sirt1 by LKB1 (Fig. S2*A* and Fig. 2*A*). In the presence of ATP, LKB1 directly phosphorylated full-length Sirt1 (Fig. 2*B*). Followed *in vitro* deacetylation assay with purified K382ac p53 peptide indicated that LKB1-directed phosphorylation is essential for Sirt1 deacetylase activity (Fig. 2*B*). No phosphorylation signal was detected in Sirt6 or Sirt7, two other nucleus-localized members of sirtuin family, suggesting the specificity of phosphorylation of Sirt1 by LKB1 (Fig. S2*B*).

To identify the LKB1 phosphorylation site(s) on Sirt1, we analyzed the phosphorylated Sirt1 with mass spectrometry. As shown in Fig. S2, *C*–*E*, three residues (Ser615, Ser669, and Ser732) were identified, which were all located at the C-terminal domain of Sirt1 (Table 1). We then created Sirt1 mutants by replacing each of or all the three serine residue(s) with alanine and performed *in vivo* and *in vitro* kinase assays. Mutation of any of these three serine residues moderately reduced the phosphorylation level of Sirt1 and replacement of all three serine residues with alanine dramatically diminished Sirt1 phosphorylation (Fig. 2*C*), suggesting that they are the major LKB1 phosphorylation sites on Sirt1. Consistently, when *in vitro* kinase assay demonstrated that Sirt1-3A mutant could not be phosphorylated by LKB1, it showed much weakened phosphorylation in resveratrol-treated cells (Fig. 2*D*, Fig. S2, *F* and *G*). In addition, depletion of AMPK failed to change the phosphorylation level of wildtype and Sirt1-3A mutant (Fig. 2*E*), suggesting that Sirt1 is not a substrate of AMPK. Thus, we concluded that Sirt1 is a direct phosphorylation substrate of LKB1, and Ser615, Ser669, and Ser732 in the C terminus of Sirt1 are the main phosphorylation residues by LKB1.

**Table 1:** Mass spectrometry search parameters and data collection

Protein	Sirt1
Zenodo DOI	10.5281/zenodo.4775266
Peaklist-generating software and release version	Progenesis QI for proteomics and V4.1
Search engine and release version	lon Accounting and 4.2.7145.32390
Sequence database searched	Uniprot-human-canonical
Release date of sequence database searched	November 19, 2018
Number of entries in the database actually searched	40,834
Specificity of protease used to generate peptide	Glu-C, Sequencing Grade (Promega, Cat.# V1651)
Missed and/or nonspecific cleavages permitted	Less than 2
Fixed modifications considered	Carbamidomethyl [C] and Oxidation [M]
Variable modifications considered	Phosphoryl [STY]
Mass tolerance for precursor ions	Less than 10 ppm
Mass tolerance for fragment ions	Less than 10 ppm
Threshold score for accepting individual spectra	Greater than 4.0
Estimation of false discovery rate (FDR)	FDR is less than 4% and FDR is calculated by Progenesis QI for proteomics software
List of all peptide sequences identified	
Peptide 1	AISVKQE
Peptide 2	RTSVAGTVRKCWPNRVAKE
Peptide 3	VYSDSEDDVLSSSSCGSNSD
Precursor charge and *m/z* for each assignment	
Peptide 1	2 charge and *m/z* 427.60
Peptide 2	5 charge and *m/z* 459.84
Peptide 3	2 charge and *m/z* 1066.8501
All modifications observed	Phosphoryl
Sites of modification within each peptide clearly located	
Peptide 1	AISVKQE [3] phosphoryl S
Peptide 2	RTSVAGTVRKCWPNRVAKE [3] phosphoryl S
Peptide 3	VYSDSEDDVLSSSSCGSNSD [13] phosphoryl S
Peptide identification scores	
Peptide 1	7.18
Peptide 2	6.20

To further determine how LKB1-mediated phosphorylation influences Sirt1 deacetylase activity, we, respectively, expressed wildtype Sirt1 or the Sirt1 mutants in HEK293T cells and analyzed their activity using the fluorometric activity assay. All the mutants represented significantly reduced deacetylase activity (Fig. 2*F*), and it was wildtype Sirt1, but not the 3A mutant, that reduced the acetylation level of p53 peptide in resveratrol-treated cells (Fig. 2*G*). These were confirmed by *in vitro* deacetylation assay using purified Sirt1 and K382ac p53 peptide as a substrate. As shown in Figure 2*H*, phosphor-disabled Sirt1-3A mutant exhibited a weaker, whereas the phosphorylation-mimetic Sirt1-3D mutant possessed a much higher, deacetylase activity than wt-Sirt1. Thus, we conclude that LKB1-dependent phosphorylation at Ser615, Ser669, and Ser732 promotes Sirt1 deacetylase activity.

### LKB1-mediated phosphorylation promotes the intramolecular interaction and relieves the intermolecular inhibition of Sirt1

**Figure 3:**
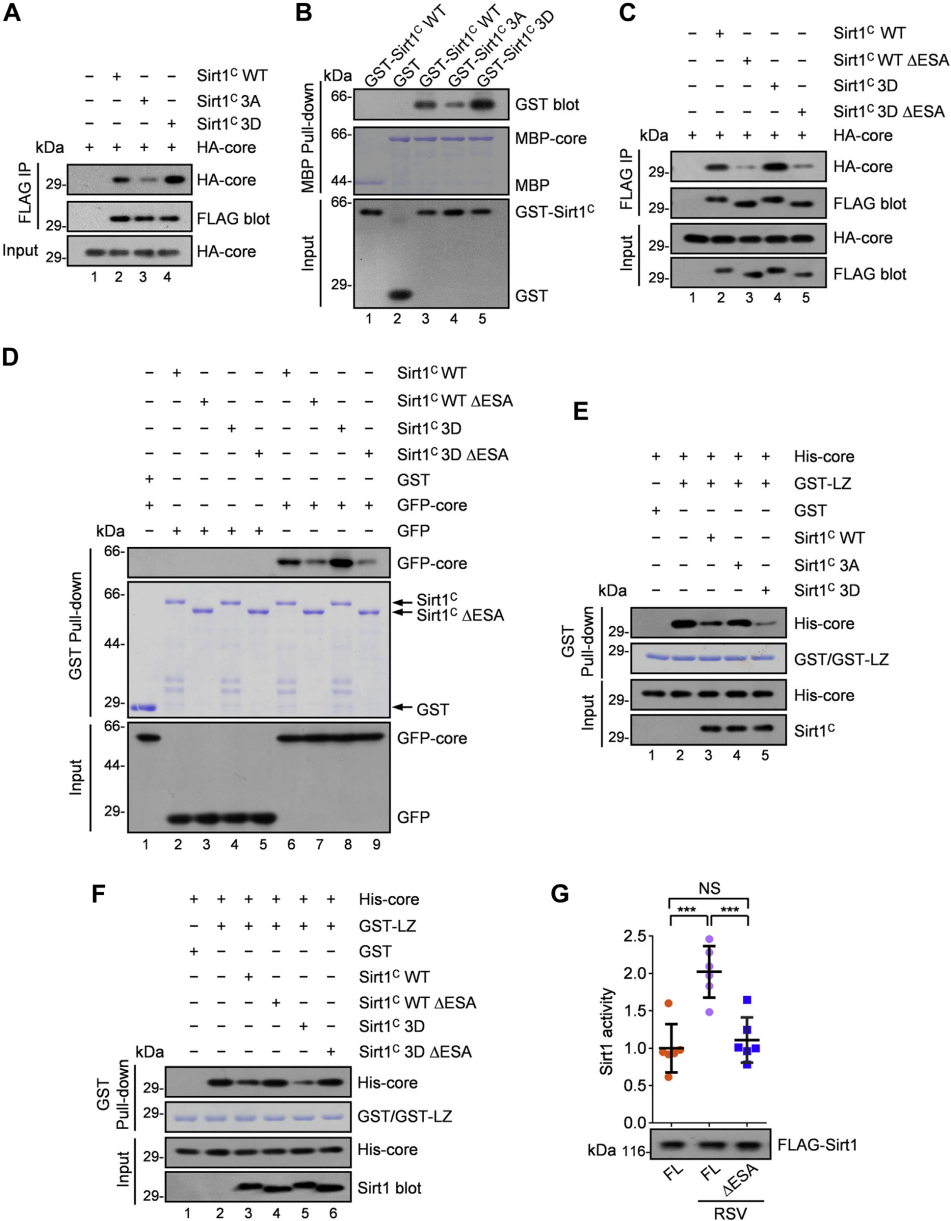
**Phosphorylation by LKB1 promotes the intramolecular interaction and relieves the intermolecular inhibition of Sirt1.***A*, coimmunoprecipitation of C terminus of Sirt1 (WT, 3A mutant, or the 3D mutant) with its deacetylase core domain. HEK293T cells were cotransfected with FLAG-Sirt1 (WT, or the mutants) of C terminus and HA-tagged core domain. Cell lysates were then subjected to immunoprecipitation with anti-FLAG antibody and immunoblotted as indicated. Representative of three independent experiments. The 3A mutation, Ser615, Ser669, and Ser732, were replaced by Ala. The 3D mutation, Ser615, Ser669, and Ser732, were replaced by Asp. *B*, MBP pull-down assay using MBP-tagged core domain of Sirt1 with GST-tagged C terminus of Sirt1 (WT, 3A mutant, or the 3D mutant) proteins. MBP-Sirt1 core domain purified from *E. coli* was incubated with eluted GST-Sirt1 WT (C terminus), GST-Sirt1 3A mutant (C terminus), or GST-Sirt1 3D mutant (C terminus). The bound fraction was analyzed by immunoblotting as indicated. Representative of three independent experiments. *C*, coimmunoprecipitation of WT or mutants of C terminus of Sirt1 with its deacetylase core domain. HEK293T cells were cotransfected with FLAG-Sirt1 WT or the mutants (ESA deletion mutant, 3D mutant, 3D mutant with ESA deletion) of C terminus and HA core. Cell lysates were then subjected to immunoprecipitation with anti-FLAG antibody and immunoblotted as indicated. Representative of three independent experiments. *D*, GST pull-down assay using GST-tagged WT or mutants of C terminus of Sirt1 with lysates of HEK293T cells that were transfected with GFP-tagged Sirt1 core domain. GST-Sirt1 WT or the mutants (ESA deletion mutant, 3D mutant, 3D mutant with ESA deletion) of C terminus purified from *E. coli* were incubated with lysates of HEK293T cells that were transfected with GFP-Sirt1 core domain. The bound fraction was analyzed by immunoblotting as indicated. Representative of three independent experiments. *E*, competitive binding experiment using GST-tagged LZ (leucine-zipper) domain of DBC1 with His-tagged Sirt1 core domain, in the presence of competing either WT or mutants of C-terminal truncations of Sirt1. We incubated 2 μg GST-LZ purified from *E. coli*, 2 μg eluted His-Sirt1 core domain, with or without 2 μg eluted WT or mutants (3A mutant or 3D mutant) of C-terminal truncation of Sirt1 for 4 h. The bound fraction was analyzed by immunoblotting as indicated. Representative of three independent experiments. *F*, competitive binding experiment using GST-tagged LZ (leucine-zipper) domain of DBC1 with His-tagged Sirt1 core domain, in the presence of competing either WT or mutants of C-terminal truncations of Sirt1. We incubated 2 μg GST-LZ purified from *E. coli*, 2 μg eluted His-Sirt1 core domain, with or without 2 μg eluted WT or mutants (ESA deletion mutant, 3D mutant, 3D mutant with ESA deletion) of C-terminal truncation of Sirt1 for 4 h. The bound fraction was analyzed by immunoblotting as indicated. Representative of three independent experiments. *G*, the activity of Sirt1 full-length or deletion mutant that was quantified by using a fluorophore-conjugated acetylated p53 peptide. HEK293T cells were infected with lentivirus-based particles expressing FLAG-Sirt1 full length or the mutant for 12 h. After 48 h, cells were treated with 25 μM resveratrol for 6 h. FLAG-Sirt1 proteins were immunoprecipitated by anti-FLAG and eluted with 3×FLAG peptide. Then 50 ng eluted FLAG-Sirt1 was incubated with 1 mM of NAD^+^ and 200 μM fluorescently labeled acetylated p53 peptide in Sirt1 assay buffer at 37 °C for 30 min, and the reaction was stopped with developer solution containing 2 mM nicotinamide. Sirt1 activity was assessed by measuring the fluorescent emission at 460 nm, following excitation at 360 nm. Data represent mean ± SD. The experiment was repeated six times independently. Statistical significance was determined by Dunnett's multiple comparisons test. NS (not significant) indicates *p* > 0.05. ∗∗∗*p* (FL, FL RSV) = 0.0001. ∗∗∗*p* (FL, FL ΔESA) = 0.0004. ΔESA, ESA region deletion; 3D mutation, Ser615, Ser669, and Ser732 were replaced by Asp; 3D ΔESA mutation, 3D mutant with ESA region deletion; Core, core domain, aa 234 to 510; ESA, essential for Sirt1 activity, aa 641 to 665 in human Sirt1 cDNA; FL, full length; LZ, leucine zipper, aa 243 to 264 of DBC1; MBP, maltose-binding protein; RSV, resveratrol; Sirt1^C^, C terminus, aa 511 to 747; WT ΔESA, WT with ESA region deletion.

The ESA region (essential for Sirt1 activity, aa 641–665) at the C terminus is indispensable for Sirt1 activation by interacting with the core domain of Sirt1 (60) (Fig. S3*A*). DBC1 (Sirt1 endogenous inhibitor) competes with ESA to bind the core domain *via* its leucine zipper (LZ, aa 243–264) domain (13, 14, 60). Because all the three phosphorylation residues by LKB1 we identified are located in the C terminus, we postulated that phosphorylation at these sites may affect the interaction between the ESA region and the core domain. To verify this, we constructed and cotransfected plasmids containing Sirt1 C terminus and core domain only. Immunoprecipitation and pull-down assays indicated that the core domain associated with more the C-terminal with the phosphomimetic 3D mutation and less the C terminal with the phosphor-disabled 3A mutation, than WT C terminus (Fig. 3, *A* and *B*, and Fig. S3*B*). Deletion of ESA abolished the interaction of either the WT C terminal or the 3D C terminal with the deacetylase core domain (Fig. 3, *C* and *D*). These results suggest that phosphorylation by LKB1 enhances the intramolecular interaction between the C-terminal domain and the core domain of Sirt1.

We next examined the effect of LKB1-dependent phosphorylation on the binding of Sirt1 to its inhibitor DBC1. The purified recombinant LZ domain of DBC1 was used to pull down the core domain of Sirt1. Apparently, the DBC1 LZ domain showed a strong association with the Sirt1 core domain, which could be decreased by coincubation in the assay of the C terminus of Sirt1 (Fig. 3*E*). Intriguingly, coincubation of the 3D C terminus of Sirt1 reduced the association even more, whereas coincubation of the 3A C terminus showed no effect on the association (Fig. 3*E*). On lack of ESA, both WT C terminus and the 3D C terminus of Sirt1 lost their function to prevent DBC1 from interacting with the core domain of Sirt1 (Fig. 3*F*).

In addition to the intramolecular core domain, the ESA region can also enhance the binding between Sirt1 and its substrates (60) (Fig. S3*C*). To examine the binding capacity of Sirt1 mutants to its substrate peptide, aliquots of 293T cell lysates from transient transfection to wildtype FLAG-Sirt1 and various mutants were incubated with the acetylated p53 peptide followed by extensive washes and Western blotting analyses. We found that the p53 peptide bound more Sirt1-3D and less Sirt1-3A (Fig. S3*D*) and the ESA region was required for the binding (Fig. S3, *E* and *F*). At last, we confirmed that the ESA region is necessary for resveratrol-stimulated Sirt1 activation (Fig. 3*G*). Thus, we conclude that LKB1-dependent phosphorylation activates Sirt1 through promoting its intramolecular interaction, which releases Sirt1 from DBC1 binding for concomitant substrate binding.

### Resveratrol promotes the binding capacity of LKB1 and Sirt1

**Figure 4:**
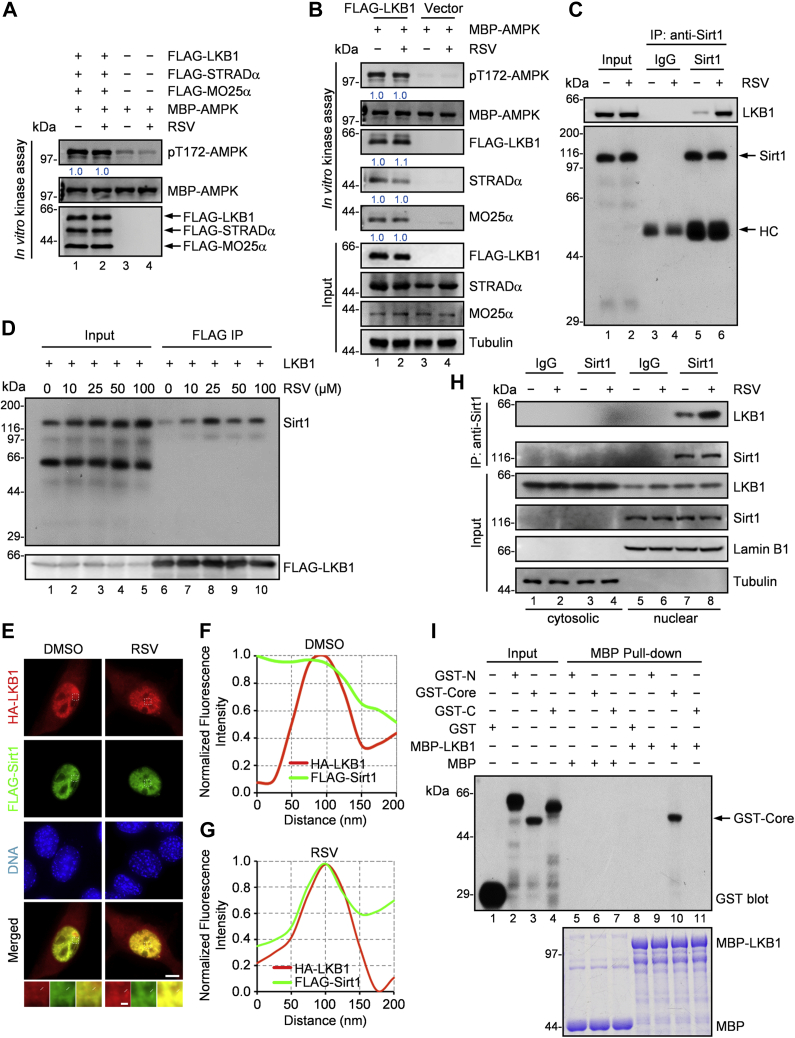
**Resveratrol promotes the binding affinity of LKB1 and Sirt1.***A*, *in vitro* kinase assay using eluted LKB1, STRADα, MO25α, and purified MBP-AMPK protein. FLAG-LKB1 plasmid, FLAG-STRADα plasmid, or FLAG-MO25α plasmid was transfected into HEK293T cells separately. FLAG-tagged proteins were immunoprecipitated from respective lysates with anti-FLAG antibody and eluted by 3×FLAG peptide. We incubated 100 ng FLAG-LKB1, 100 ng FLAG-STRADα, 100 ng FLAG-MO25α, and 2 μg purified MBP-AMPK protein with or without 25 μM resveratrol (RSV) in 40 μl kinase assay buffer at 30 °C for 30 min. Samples were subjected to immunoblotting as indicated. Representative of three independent experiments. *B*, *in vitro* kinase assay using eluted LKB1 complex with purified MBP-AMPK protein. FLAG-LKB1 plasmid was transfected into HEK293T cells and cells were treated with 25 μM RSV for 6 h. FLAG-LKB1 proteins were immunoprecipitated by anti-FLAG and were eluted with 3×FLAG peptide. And 100 ng eluted protein and 2 μg purified MBP-AMPK protein were incubated in 40 μl kinase assay buffer at 30 °C for 30 min. Samples were subjected to immunoblotting as indicated. The LKB1 kinase activity was remarked by phosphorylation of Thr172 site of AMPK. The bound fraction about FLAG-LKB1 was analyzed by using anti-STRADα antibody and anti-MO25α antibody. Representative of three independent experiments. *C*, immunoprecipitation of Sirt1 with LKB1 in RSV-treated C2C12 myotubes. C2C12 myoblast cells were grown in DMEM with 2% horse serum for 4 days. Cells were treated with 25 μM RSV for 6 h. Lysates were subjected to immunoprecipitation using antibody against Sirt1. The precipitates were examined by immunoblotting with anti-LKB1 antibody. *D*, immunoprecipitation of FLAG-LKB1 with Sirt1 in RSV-treated HEK293T cells. HEK293T cells were infected with lentivirus-based particles expressing FLAG-LKB1 and then were treated with 0 to 100 μM RSV for 6 h. FLAG-LKB1 proteins were immunoprecipitated by anti-FLAG, and the bound fraction was analyzed by using anti-Sirt1 antibody. Representative of three independent experiments. *E*, immunofluorescent images of subcellular distribution about LKB1 and Sirt1. HA-LKB1 plasmid and FLAG-Sirt1 plasmid were cotransfected into C2C12 cells, and cells were then treated with 25 μM RSV for 30 min. Cells were stained with anti-mouse-HA antibody (*red*), anti-rabbit-FLAG antibody (*green*), and DAPI (*blue*). Scale bar, 5 μm. The scale bar for zoom magnification panels, 0.2 μm. *F* and *G*, fluorescence intensity distribution for HA-LKB1 and FLAG-Sirt1 imaging of an arbitrary 200-nm line (as *white line* indicated) was calculated and plotted. The fluorescence intensity was normalized to the highest value in the region to facilitate the comparison. *H*, coimmunoprecipitation of LKB1 with Sirt1 in the nuclear or cytoplasmic fractions from treated HEK293T cells with or without RSV. Sirt1 was immunoprecipitated using anti-Sirt1, and the precipitates were analyzed using anti-LKB1. Representative of three independent experiments. *I*, GST-Sirt1-N (N terminus, aa 1–233), GST-Sirt1-Core (deacetylase core, aa 234–510), and GST-Sirt1-C (C terminus, aa 511–747) proteins were purified from *E. coli*. Then each of the purified proteins was incubated with recombinant MBP-LKB1, and a pull-down assay for Sirt1 fragments was performed using specific GST antibody. Representative of three independent experiments.

To further investigate the mechanism by which LKB1 activates Sirt1 in response to resveratrol stimulation, we first examined whether resveratrol directly activates LKB1. We performed an *in vitro* kinase assay by incubating immunoprecipitated FLAG-tagged LKB1, STRADα, and MO25α, with recombinant AMPK complex, in the presence of resveratrol. Adding resveratrol to the kinase reaction showed no effect on the level of AMPK phosphorylation at Thr172 (Fig. 4*A*). We then used LKB1 complex from resveratrol-treated cells and carried out similar experiments. Still, no detectable increase in AMPK phosphorylation was observed (Fig. 4*B*). These data suggest that resveratrol is not able to raise the kinase activity of LKB1 either *in vitro* or in cell.

We next tested whether resveratrol could promote the binding between LKB1 and Sirt1. With coimmunoprecipitation assay, we found that treatment of cells with resveratrol dose dependently increased the interaction between LKB1 and Sirt1 (Fig. 4, *C* and *D*, Fig. S4, *A*–*C*). We also observed the increased interaction between LKB1 and Sirt1 with a parallel rise in phosphorylation of Sirt1 at various time points (Fig. S4*D* and Fig. 1*A*). Meanwhile, no LKB1 was detected in the FLAG-Sirt6 or FLAG-Sirt7 immunoprecipitates (Fig. S4*E*), indicating the specific binding between LKB1 and Sirt1. Consistent with the effect of resveratrol, treatment of cells with another Sirt1 activator SRT1720 also upregulated the interaction between LKB1 and Sirt1 (Fig. S4*F*). We further analyzed the subcellular distribution of LKB1 and Sirt1 in different cell lines. Compared with the very weak colocalization in untreated cells, LKB1 in resveratrol-treated cells showed strong colocalizations with Sirt1 in the nucleus (Fig. 4, *E*–*G*). We then performed coimmunoprecipitation analysis in the nuclear fraction and cytoplasmic fraction, respectively. Immunoprecipitation of Sirt1, one of the sirtuin proteins primarily resided in nucleus, coprecipitated LKB1 in the nuclear fraction rather than in the cytoplasmic fraction, suggesting that LKB1 can associate with Sirt1 in the nucleus (Fig. 4*H*). In addition, resveratrol treatment markedly enhanced the coimmunoprecipitation of LKB1 with Sirt1 (Fig. 4*H*). Therefore, resveratrol can promote the interaction between LKB1 and Sirt1 in the cell nucleus. However, our enzymatic assay to assess the direct interaction between LKB1 and Sirt1 indicated that addition of resveratrol did not alter the kinetics of LKB1 kinase activity (Fig. S4, *G* and *H*), suggesting that the resveratrol-elicited LKB1 interaction with Sirt1 seen in the nucleus is regulated by the signaling cascade. We further evaluate whether the interaction between LKB1 and the Sirt1 seen in resveratrol treated-cells can be recapitulated using different domains and/or fragments. To this end, aliquots of purified recombinant GST-Sirt1 proteins were used as affinity matrix to absorb recombinant MBP-LKB1. As shown in Figure 4*I*, GST-Sirt1-core, but not GST-Sirt1-N or GST-Sirt1-C, absorbed LKB1 protein (Fig. 4*I*; lane 10). To test whether the Sirt1-core fragment interacts with LKB1, 293T cells were transiently transfected to express various GFP-Sirt1 deletion mutants and FLAG-LKB1. As shown in Fig. S4*I*, GFP-Sirt1-core selectively absorbed LKB1 (lane 7). Together, these data support the notion that resveratrol promotes the binding of LKB1 to Sirt1 without affecting LKB1 kinase activity. The deacetylase core region of Sirt1 provides a docking site for LKB1, enabling LKB1 to phosphorylate at the C terminus of Sirt1.

### LKB1-mediated Sirt1 activation increases mitochondrial biogenesis and respiration

**Figure 5:**
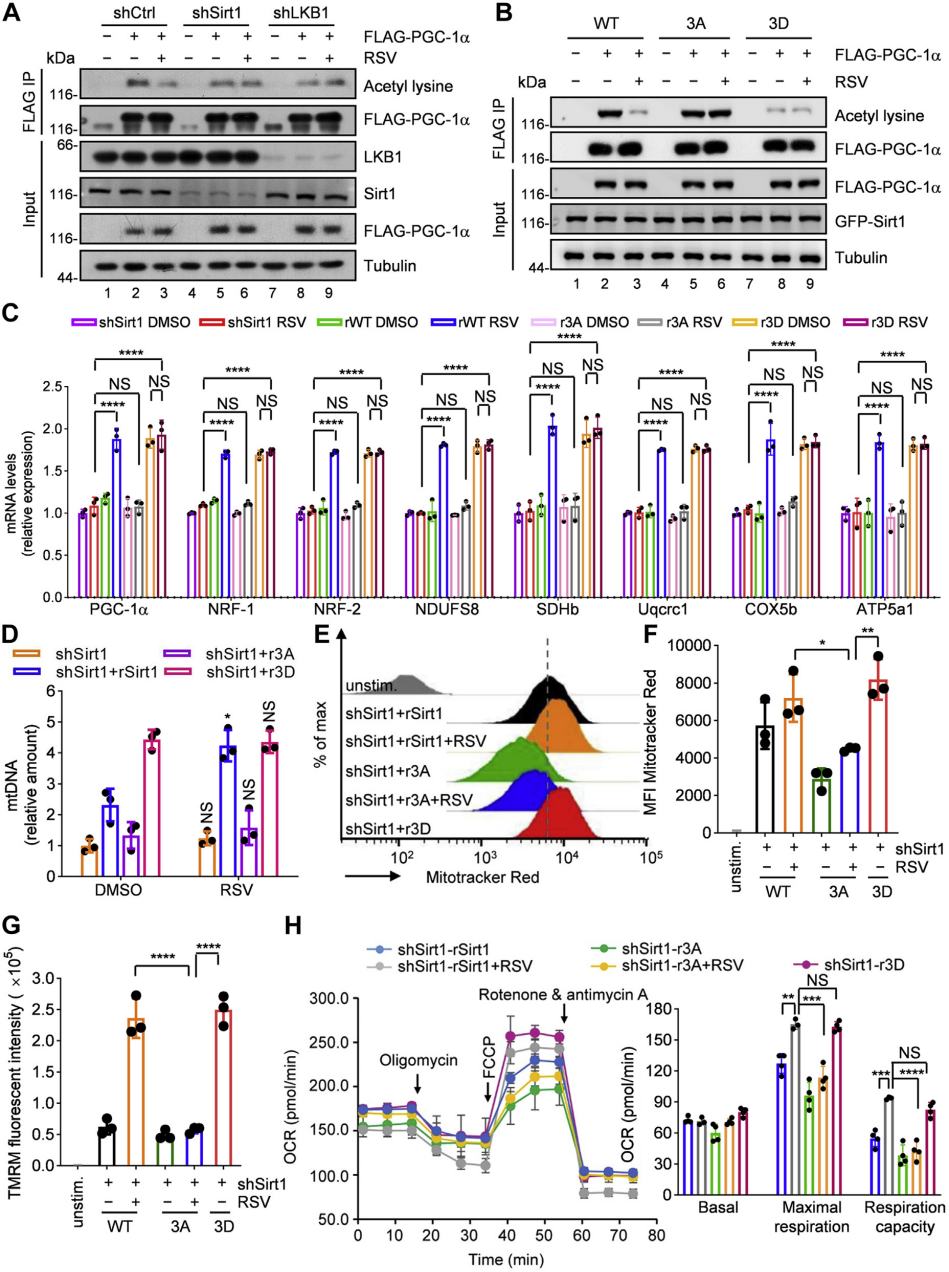
**LKB1-mediated Sirt1 activation promotes mitochondrial function.***A*, deacetylation of PGC-1α by Sirt1 in resveratrol-treated LKB1-depleted HEK293T cells. HEK293T cells were infected with lentivirus-based particles expressing shRNA control, Sirt1 shRNA, or LKB1 shRNA for 12 h, and 36 h later cells were infected with virus particles expressing FLAG-PGC-1α for 12 h. After 36 h, cells were treated with 25 μM resveratrol for 6 h. FLAG-PGC-1α proteins were immunoprecipitated by anti-FLAG and immunoblotted with anti-acetylated-lysine antibody. Representative of three independent experiments. *B*, deacetylation of PGC-1α by Sirt1 in resveratrol-treated Sirt1-depleted HEK293T cells expressing WT Sirt1, 3A mutant, or 3D mutant. HEK293T cells stably expressing Sirt1 shRNA were coinfected with lentivirus-based particles expressing FLAG-PGC-1α and lentivirus-based particles expressing GFP-Sirt1 WT, the 3A mutant, or the 3D mutant for 12 h, and 48 h later cells were treated with 25 μM resveratrol for 6 h. FLAG-PGC-1α proteins were immunoprecipitated by anti-FLAG and immunoblotted with anti-acetylated-lysine antibody. *C*, PGC-1α, NRF-1, NRF-2, NDUFS8, SDHb, Uqcrc1, Cox5b, ATP5a1 mRNA were analyzed by means of quantitative PCR in resveratrol-treated Sirt1-depelted C2C12 cells infected with lentivirus-based particles expressing WT, 3A mutant, or 3D mutant of Sirt1. Relative expression values were normalized to untreated cells. Data represent mean ± SD. The experiment was repeated three times independently. Statistical significance was determined by Tukey's multiple comparisons test. NS (not significant) indicates *p* > 0.05. ∗∗∗∗*p* < 0.0001. WT group indicates WT Sirt1 rescue in Sirt1-depleted cells. The 3A group indicates Sirt1 3A mutant (Ser615, Ser669, and Ser732 were replaced by Ala) rescue in Sirt1-depleted cells. The 3D group indicates Sirt1 3D mutant (Ser615, Ser669, and Ser732 were replaced by Asp) rescue in Sirt1-depleted cells. *D*, mitochondrial content analyzed by means of quantitative PCR in C2C12 cells treated with 25 μM resveratrol. Relative expression values were normalized to untreated cells. Data represent mean ± SD. The experiment was repeated three times independently. Statistical significance was determined by unpaired two-tailed *t* test. NS (not significant) indicates *p* > 0.05. The *p* (shSirt1, shSirt1 RSV) = 0.2497, ∗*p* (shSirt1+rSirt1, shSirt1+rSirt1 RSV) = 0.0103, *p* (shSirt1+r3A, shSirt1+r3A RSV) = 0.5695, *p* (shSirt1+r3D, shSirt1+r3D RSV) = 0.8046. The shSirt1+rSirt1 group indicates WT Sirt1 rescue in Sirt1-depleted cells. The shSirt1+r3A group indicates Sirt1 3A mutant rescue in Sirt1-depleted cells. The shSirt1+r3D group indicates Sirt1 3D mutant rescue in Sirt1-depleted cells. *E*, mitochondrial density in C2C12 cells was measured by flow cytometry using MitoTracker Red CMXRos. *F*, the relative change in the mean fluorescence intensity of MitoTracker in panel *E*. Data represent mean ± SD. The experiment was repeated three times independently. Statistical significance was determined by Tukey's multiple comparisons test. ∗*p* = 0.0252, ∗∗*p* = 0.0026. *G*, in C2C12 cells used in panel *E*, mitochondrial membrane potential was measured by ImageStream mark ii flow cytometer using tetramethyl rhodamine methyl ester (TMRM). Data represent mean ± SD. The experiment was repeated three times independently. Statistical significance was determined by Tukey's multiple comparisons test. ∗∗∗∗*p* < 0.0001. *H*, in C2C12 cells used in panel *E*, seahorse assays were conducted. Oxygen consumption rate (OCR) over time (*left panel*) and OCR in different stages of the measurement (*right panel*) are shown. Data represent mean ± SD. Statistical significance was determined by Tukey's multiple comparisons test. NS (not significant) indicates *p* > 0.05. In maximal respiration measurement, ∗∗*p* = 0.0026, ∗∗∗*p* = 0.0001. In respiration capacity measurement, ∗∗∗*p* = 0.0001, ∗∗∗∗*p* < 0.0001. RSV, resveratrol.

PGC-1α is a master mediator of the metabolic effects of resveratrol (30, 61). It is a coactivator that controls the transcription of genes involved in mitochondrial function (10, 35). Since Sirt1 promotes mitochondrial function through deacetylation and activation of PGC-1α (10, 35), we speculated that LKB1-dependent Sirt1 activation functions in mitochondrial homeostasis. To verify this hypothesis, we first explored the role of LKB1-mediated Sirt1 phosphorylation in regulating PGC-1α transcriptional activity. Both LKB1 knockdown and Sirt1 knockdown dramatically reduced the resveratrol-stimulated PGC-1α deacetylation (Fig. 5*A* and Fig. S5*A*), indicating that LKB1 acts upstream of coactivator transcription. Expression of wildtype Sirt1, but not the phosphorylation-disabled 3A mutant, reversed the effect of LKB1 depletion or Sirt1 depletion. In addition, expression of phosphorylation-mimetic Sirt1-3D mutant significantly reduced PGC-1α deacetylation, consistent with the expression of wt-Sirt1 in resveratrol-treated cells (Fig. 5*B*, Fig. S5, *B* and *C*). These data support the notion that the phosphorylation of Sirt1 by LKB1 is required to mediate deacetylation of PGC-1α by activated Sirt1.

As a coactivator, PGC-1α induces transcription of various downstream genes, which comprise multiple genes in regulating mitochondrial function (28). Consistent with previous findings, resveratrol treatment actually increased mRNA expression of a number of genes downstream of PGC-1α including transcription factors responsible for stimulating mitochondrial biogenesis (NRF-1 and NRF-2) and components of mitochondrial electron transport chain (NDUFS8, SDHb, Uqcrc1, COX5b, ATP5a1) (Fig. S5*D*), whereas LKB1 or Sirt1 knockdown prevented resveratrol-induced augmentation of the related mRNA levels (Fig. S5, *D* and *E*). Next, we checked the restoration of Sirt1 depletion-induced inhibition of transcription by Sirt1-3A and Sirt1-3D. As expected, only the expression of wt-Sirt1, but not phosphorylation-disabled Sirt1-3A mutant, reversed the effect of Sirt1 knockdown on the transcription of genes related to mitochondrial biogenesis and respiration. Expression of phosphorylation-mimetic Sirt1-3D mutant reversed the effect of Sirt1 knockdown, consistent with the expression of wt-Sirt1 in resveratrol-treated cells (Fig. 5*C* and Fig. S5*F*). Taken together, these results suggest that phosphorylation of Sirt1 by LKB1 is essential for the transcription of genes related to mitochondrial function by activating PGC-1α.

To further confirm the function of LKB1-mediated Sirt1 phosphorylation in mitochondrial biogenesis, we first measured the mitochondrial DNA (mtDNA) content in LKB1-depleted or Sirt1-depleted C2C12 myotubes treated with resveratrol. As shown in Fig. S5, *G* and *H*, resveratrol treatment significantly increased the mitochondrial DNA content, whereas LKB1 or Sirt1 knockdown reversed this effect. Of note, expression of wt-Sirt1, but not Sirt1-3A mutant, rescued the mtDNA content in Sirt1-depleted cells. Expression of phosphorylation-mimetic Sirt1-3D mutant significantly increased mtDNA content, consistent with the expression of wt-Sirt1 in resveratrol-treated cells (Fig. 5*D* and Fig. S5*F*). Furthermore, mitochondrial density was measured by flow cytometry using MitoTracker Red CMXRos, a fluorescent probe that specifically labeled mitochondrial organelle. In Sirt1-depleted cells, expression of wt-Sirt1, but not the phosphorylation-disabled Sirt1-3A mutant, increases mitochondrial density with resveratrol treatment. Of interest, expression of phosphorylation-mimetic Sirt1-3D mutant evidently augmented mitochondrial density, consistent with the expression of wt-Sirt1 in resveratrol-treated cells (Fig. 5, *E* and *F*, and Fig. S5*I*). To establish mitochondria quality control, mitochondrial membrane potential was measured with a fluorescent probe, tetramethyl rhodamine methyl ester (28). In Sirt1-depleted cells, expression of wt-Sirt1, but not the phosphorylation-disabled Sirt1-3A mutant, increases mitochondrial membrane potential with resveratrol treatment. Expression of phosphorylation-mimetic Sirt1-3D mutant evidently augmented mitochondrial membrane potential, consistent with the expression of wt-Sirt1 in resveratrol-treated cells (Fig. 5*G*, Fig. S5, *I* and *J*). Next, we tested the function of LKB1-dependent Sirt1 phosphorylation in mitochondrial respiration. Since phosphorylation-disabled Sirt1-3A mutant inhibited the transcription of components of mitochondrial electron transport chain, it is expected that the oxygen consumption rate could be enhanced when Sirt1 is phosphorylated by LKB1. Indeed, as in the Sirt1-depleted cells, resveratrol produced substantial increases in maximal respiration (FCCP-induced) and respiration capacity when re-expressing wt-Sirt1. Strikingly, none of the significant increases in mitochondrial function were observed in Sirt1-3A-expressing cells (Fig. 5*H* and Fig. S5*I*). The beneficial effects of resveratrol were clearly evident in Sirt1-3D-expressing cells, confirming that phosphorylation of S615, S669, and S732 was required for LKB1-mediated regulation of Sirt1 in mitochondrial function (Fig. 5*H* and Fig. S5*I*). Together, these results suggest that LKB1-dependent phosphorylation of Sirt1 is crucial for mitochondrial biogenesis and respiration through the activation of PGC-1α.

## Discussion

**Figure 6:**
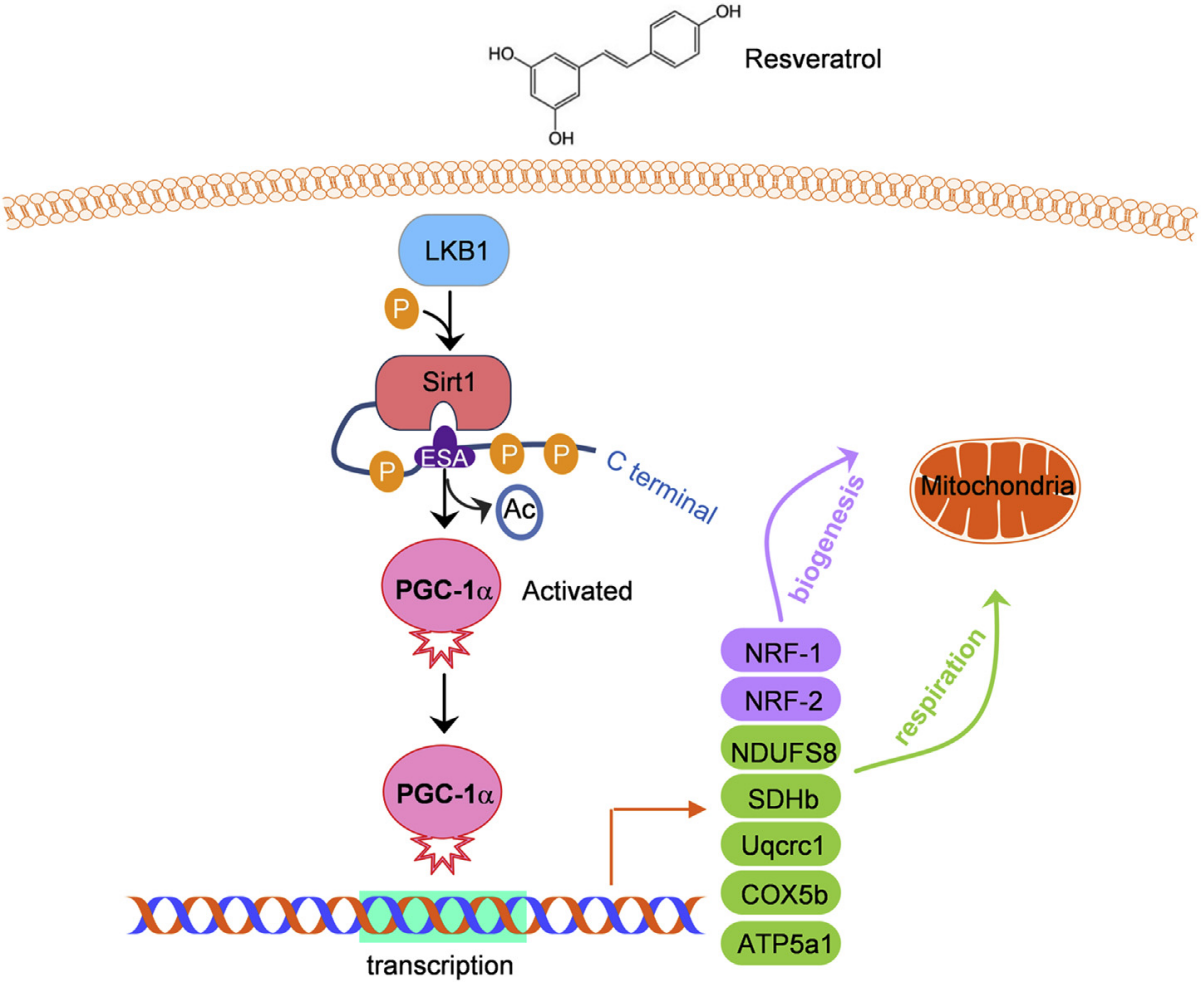
**Schematic model for resveratrol-stimulated LKB1-mediated phosphorylation and activation of Sirt1 in mitochondrial biogenesis and respiration.** Resveratrol promotes the binding between LKB1 and Sirt1, which drives LKB1 to directly phosphorylate Sirt1 at three different residues in the C-terminal domain. LKB1-dependent phosphorylation promotes the intramolecular interaction of Sirt1 and activates its deacetylase activity. Functionally, activated Sirt1 increases mitochondrial biogenesis and respiration through deacetylation and activation of PGC-1α, a master regulator of mitochondrial function.

The longevity-associated Sirt1 deacetylase orchestrates cell plasticity control and organ function. The natural product resveratrol has been recognized as a Sirt1 activator for a long time, but controversy exists around the mechanism of Sirt1 activation. Here, our identification of the resveratrol-elicited LKB1-Sirt1 signaling axis uncovered a new regulatory mechanism by which activation of LKB1-mediated phosphorylation of Sirt1 by resveratrol promotes the activity of Sirt1 for control of genomic stability (Fig. 6). It would be of great interest, in follow-up studies, to characterize additional substrates of LKB1 in protection against metabolic stress in mammals.

Resveratrol was discovered as an activator of Sirt1 by using fluorophore-tagged substrates, and it activates Sirt1 to deacetylate fluorophore-conjugated substrates but not native substrates (62, 63, 64), suggesting that resveratrol activates Sirt1 indirectly *in vivo* and it may activate Sirt1 *via* an upstream target. LKB1 acts as an evolutionarily conserved regulator of cellular energy metabolism and functions as the major upstream kinase to phosphorylate AMPK and 12 other AMPK-related kinases (65, 66). An important feature of this model is the identification of LKB1 function in the nucleus elicited by resveratrol, where Sirt1 is localized, can quickly and efficiently phosphorylate Sirt1, and then activated Sirt1 prompts genes transcription by deacetylating co-activator PGC-1α. Identification of the LKB1-Sirt1 pathway in mitochondrial function would facilitate our better understanding of cell renewal and energy metabolism. An important example might be hematopoietic stem cell (HSC) homeostasis, in which LKB1 balances proliferation and quiescence in HSCs by regulating cell survival, cell cycle, and mitochondrial function in an AMPK-, mTORC1-, FoxO-independent mechanism (67, 68, 69). However, the specific effectors of LKB1 in HSCs have yet to be defined (70). Our present study delineates the cellular function of LKB1 in mitochondrial homeostasis. These results explain how LKB1 serves as a genome guardian by orchestrating cell renewal and mitochondrion function during cell division. Furthermore, LKB1-mediated Sirt1 activation may also play physiological roles in the context of antiaging and anticancer. Calorie restriction has been demonstrated to extend the life span of *Saccharomyces cerevisiae via* the activity of Sir2 (71). Overexpression of Sirt1, the mammalian homolog of Sir2, has been described to protect mice from aging and cancer (72, 73). As the most potential activator of Sirt1, resveratrol induces gene expression patterns that resembled those induced by calorie restriction and delays aging-related phenotypes in mice (26). Therefore, we propose that LKB1-mediated Sirt1 activation may respond to energy restriction (such as glucose starvation) and play roles in aging and cancer, which needs further study.

Sirt1 has been shown to play a number of similar roles as AMPK, including the ability to respond to metabolic stress, prompt mitochondrial function, regulate glucose homeostasis, and control the activity of critical transcriptional regulators such as PGC-1α, p300, and FOXOs (74). In line with this, the beneficial effects of resveratrol have also been identified to involve the activation of Sirt1 and AMPK (61, 75, 76). Some reports suggest that resveratrol primarily activates AMPK, potentially by inhibiting PDEs, ATPase, or complex III (77, 78, 79), and then AMPK activates Sirt1 indirectly by increasing the cellular level of NAD^+^ (58). Alternatively, resveratrol may first activate Sirt1, based on the results that AMPK cannot be activated, and its beneficial effect on mitochondrial metabolism is seriously attenuated in Sirt1 knockout mice, whereas the results from Sirt1 overexpressed mice are reversible (28). Furthermore, resveratrol is likely to exert different effects depending on the concentration used, the timing of the treatment, and the cell type in question. At high concentrations (100–300 μM), resveratrol activates AMPK by decreasing energy and increasing the AMP/ATP or ADP/ATP ratios (77, 78, 79). At low concentrations (<50 μM), resveratrol appears to activate AMPK without decreasing energy (61, 75, 76). Cell culture experiments performed with hepatocytes demonstrated that Sirt1 is required for resveratrol to induce phosphorylation of AMPK and increase mitochondrial function. However, resveratrol promotes mitochondrial function in both wildtype and Sirt1 knockout mice (28), suggesting the complex interplay between different situations. Thus, it is difficult to untangle the epistasis of Sirt1 and AMPK, but clarifying their interplay can help us better understand the activation mechanism of Sirt1. We identified here that Sirt1 clearly was activated in resveratrol-treated (25 μM, 6 h) AMPK-depleted or AMPK-inhibited cells, indicating an independence from AMPK activity. Inversely, AMPK activity was significantly reduced in resveratrol-treated LKB1-depleted cells, whereas Sirt1 knockdown had no effect (data not shown), consistent with previous findings that resveratrol-stimulated AMPK activity in neurons depended on LKB1 activity but did not require the NAD^+^-dependent protein deacetylase Sirt1 (75). Our findings present a novel mechanism of Sirt1 activation by posttranslational modification mediated by LKB1 that can be physiologically regulated.

Identification of the phosphorylation sites allows us to investigate the molecular nature of Sirt1 activation by LKB1. Utilizing the Sirt1 phosphorylation-disabled and phosphorylation-mimicking mutants, our analyses revealed that phosphorylation is essential and sufficient for the activation of Sirt1 deacetylase. In addition, this phosphorylation-based activation involves promotion of the intramolecular interaction of Sirt1. Structurally, Sirt1 possesses a long disordered C-terminal domain, which is indispensable for Sirt1 deacetylase activity (60, 80, 81). Phosphorylation of Sirt1 by LKB1 induces the binding of the C terminus to the deacetylase core domain, possibly through a conformational change in the protein. Thus, our findings may represent a general mechanism for Sirt1 activation in which C-terminal phosphorylation released DBC1 binding to the deacetylase core domain of Sirt1 by which DBC1 inhibition is liberated (13, 14). Although high resolution of the 3D structure of full-length mammalian Sirt1 is not yet available, biochemical study showed that the ESA (essential for Sirt1 activity) region located in the C terminus of Sirt1 competes with DBC1 to interact with the deacetylase core, thereby eliciting Sirt1 activity (60). In addition, the mutation to 615A seems to inhibit Sirt1 activity more than the other two (Fig. 2*F*), which suggests that S615 is more important in regulating Sirt1’s activity. Since these three sites influence the binding of DBC1 to Sirt1’s core domain, and S615 is closer to the core domain, we propose that S615 may have a greater blocking effect on DBC1 than the other two sites, which needs to be identified in future.

Our present study revealed that the phosphorylation of Sirt1 at S615, S669, and S732 by LKB1 may regulate the electrostatic interactions between the C-terminal ESA region and deacetylase core domain, thereby dissociating Sirt1 from DBC1 and releasing Sirt1 activity. Our current analysis suggests that resveratrol treatment promotes the binding of LKB1 to Sirt1 *in vivo*. However, resveratrol exhibits no direct effect on LKB1-Sirt1 interaction *in vitro* (Fig. S4, *G* and *H*). The inconsistency between *in vivo* and *in vitro* assays suggests that signaling pathways regulating the binding of LKB1 and Sirt1 exist in resveratrol-treated cells. Future investigation using proximity ligation would enable us to consolidate the resveratrol-elicited Sirt1 activation into signaling cascades underlying LKB1-regulated mitochondrion activity and cellular plasticity control.

In sum, we report a previously unrecognized molecular mechanism that underlies Sirt1 activation and define a signaling axis that integrates LKB1 phosphorylation to mitochondrion dynamics. It is maybe helpful for us to understand and treat Sirt1 correlated ageing-related diseases.

## Experimental procedures

### Plasmid constructs

Site-specific mutants or deletion mutants of FLAG-Sirt1, HA-LKB1, FLAG-STRADα, GFP-MO25α, HA-p53, and GFP-p300 HAT (aa 3583–5280) were generated by PCR-based, site-directed mutagenesis kit from Vazyme (C112) according to the manufacturer’s instructions. GST-, His-, and MBP-tagged Sirt1 were generated by subcloning human Sirt1 cDNA into pGEX-6p-1, pET28a, and pMal-c2X, respectively. Mammalian coexpression plasmid HA-LKB1-STRADα was generated by inserting HA-LKB1 from the corresponding vector into MCS of pIRES2-ZsGreen1 vector and replacing ZsGreen1 coding frame with STRADα. Baculovirus expressed His-tagged Sirt1 deacetylase was generated by inserting human Sirt1 (aa 193–747, Abcam, recombinant human Sirt1 protein, ab101130) into pFastBac plasmid. Baculovirus-based coexpression plasmid His-LKB1-STRADα-MO25α was a gift from laboratory of Daan M. F. van Aalten (42). The p53-GST (aa 373–385) construct was obtained by inserting human p53 (aa 373–385) into pCDF-pylT. AAG codon corresponding to 382 site was mutated to TAG, in order to generate p53-382TAG-GST plasmid. ACKRS3 and pCDF-pylT plasmids were gifts from laboratory of J. Chin. For lentivirus-based protein expression, wildtype or site-specific mutants of Sirt1, and LKB1 were inserted into lentivirus-based vector pLVX-FLAG. The constructed plasmid along with psPAX2 and pMD.2G was used for producing packaged virus particles.

### Cell culture, differentiation, and lentivirus infection

C2C12 myoblast cells (ATCC), HeLa cells, and HEK293T cells were maintained as monolayers in advanced Dulbecco's modified Eagle's medium (DMEM) (Invitrogen) with 10% fetal bovine serum (HyClone) and 100 units/ml of penicillin plus 100 μg/ml of streptomycin (Invitrogen). To generate C2C12 myotubes, C2C12 cells were grown in DMEM with 2% horse serum for 4 days. For lentivirus infection, C2C12 cells were infected with packaged lentivirus particles for 12 h and were continuously grown in fresh medium for 48 h. After infection, the cells were grown in DMEM with 2% horse serum for 4 days to generate myotubes. Cells were treated the vehicle (dimethyl sulfoxide [DMSO]) or 25 μM resveratrol for 6 h. To investigate the effects of various polyphenols on Sirt1 activation, differentiated C2C12 cells were treated with DMSO (vehicle), 25 μM piceatannol (Sirt1 activator), 25 μM fisetin (Sirt1 activator), 25 μM quercetin (Sirt1 activator), 25 μM resveratrol (Sirt1 activator), 1 μM EX527 (Sirt1 inhibitor), or 10 mM nicotinamide (Sirt1 inhibitor) for 6 h. For Sirt1 dephosphorylation, immunoprecipitated Sirt1 was treated with lambda protein phosphatase at 30 °C for 30 min. To increase *in vivo* K382 acetylation of p53, cells were treated with 1 μM doxorubicin for 1 h.

### Antibodies and reagents

Anti-Sirt1 (#2493, 1:1000), anti-LKB1 (#13031, 1:1000), anti-acetyl-p53 K382 (#2525, 1:1000), anti-p53 (#18032, 1:1000), anti-phospho-AMPK T172 (#2535, 1:1000), anti-AMPK (#5831, 1:1000), anti-phospho-ACC Ser79 (#11818, 1:1000), anti-ACC (#3662, 1:1000), anti-MO25α (#2716, 1:2000), anti-acetylated-lysine (#9441, 1:1000), anti-Lamin B1 (#13435, 1:1000), anti-GST-tag (#2625, 1:2000), anti-His-tag (#12698, 1:2000), anti-HA-tag (#3724, 1:2000), and anti-MBP-tag (#2396, 1:2000) antibodies were from Cell Signaling Technology. Anti-phospho-Ser/Thr (ab17464, 1:1000) and anti-α-tubulin (ab80779, 1:5000) antibodies were from Abcam. Anti-STRAD (N-13) (sc-34102) was from Santa Cruz. Anti-FLAG-tag (M2, 1:2000) antibody was from Sigma. Lambda Protein Phosphatase (P0753) was from New England Biolabs. Resveratrol, piceatannol, quercetin, fisetin, EX527, nicotinamide, NAD, N-ε-acetyl-L-lysine, SRT1720, 3xFLAG peptide, doxorubicin, phosphatase inhibitors, and protease inhibitors were from Sigma.

### RNA interference and lentivirus production

The lentivirus-based vector pLKO.1 along with psPAX2 and pMD.2G were used for producing shRNA-packaged viral particles. The nucleotide sequence for shRNA against human Sirt1 was 5′-CAGGTCAAGGGATGGTATTTA-3′ (shSirt1-h1) and the sequence for shRNA against mouse Sirt1 was 5′-CAGATCAAGAGACGGTATCTA-3′ (shSirt1-m1). The nucleotide sequence for shRNA against human LKB1 was 5′-GGGTCACCCTCTACAACATCA-3′ (shLKB1-h4) and the sequence for shRNA against mouse LKB1 was 5′- GGGTCACACTTTACAACATCA-3′ (shLKB1-m4). The nucleotide sequence for shRNA against human AMPKα1α2 and mouse AMPKα1α2 was 5′-ATGATGTCAGATGGTGAATTT-3′. The nucleotide sequence for shRNA against CAMKKβ was 5′-GTGAAGACCATGATACGTAAA-3′, the sequence for shRNA against DYRK1A was 5′-GAACCTAACACGAAAGTTTGC-3′, the sequence for shRNA against DYRK3 was 5′-CTCCACCCAGAAGACTAAATA-3′, the sequence for shRNA against JNK1 was 5′-GCCCAGTAATATAGTAGTAAA-3′, the sequence for shRNA against JNK2 was 5′-CCAGATGCTTTGTGGTATTAA-3′, and the sequence for shRNA against CK2α was 5′-AGCCATCAACATCACAAATAA-3′. For producing shRNA-packaged viral particles, constructed pLKO.1 plasmid along with psPAX2 and pMD.2G were transfected into HEK293T cells with Lipofectamine 2000 reagent (Invitrogen) for 12 h according to manufacturer’s instructions. After transfection, cells were changed to fresh medium and 48 h later the lentivirus supernatant was collected. For protein expression, constructed pLVX-FLAG or pLVX-GFP plasmid along with psPAX2 and pMD.2G were transfected into HEK293T cells with Lipofectamine 2000 reagent for 12 h. After transfection, cells were changed to fresh medium, and 48 h later the lentivirus supernatant was collected to infect C2C12 myoblasts or HeLa cells. For stable expression, the lentivirus supernatant was used to infect cells. On the following day the cells were passaged and 24 h later selected with puromycin (2 μg/ml) for 2 weeks.

### Recombinant protein expression

For acetylated protein purification, *E. coli* strain Rosetta (DE3) was transformed with pACKRS and p53-382TAG-GST plasmids simultaneously. The bacteria were cultured in lysogeny broth medium supplemented with kanamycin (50 mg/ml) and spectinomycin (50 mg/ml) to *A*_600_ of 0.7. Acetyl-lysine (AcK, 10 mM) and NAM (20 mM) were then added, the culture was incubated for 0.5 h, and protein expression was induced with IPTG (0.2 mM) at 37 °C for 4 h. The bacteria were lysed by sonication in PBS buffer (137 mM NaCl; 2.7 mM KCl; 4.3 mM Na_2_HPO_4_; 1.4 mM KH_2_PO_4_, pH 7.4) and incubated with Glutathione agarose (GE Healthcare Life Science) for 1.5 h at 4 °C. The agarose was washed three times in PBS buffer and eluted with 10 mM reduced glutathione.

GST- and His-tagged Sirt1 truncations and site mutants, MBP-AMPKα2 were produced from bacteria as described (82). Basically, the plasmids were transformed into *E. coli* strain Rosetta (DE3), and protein expression was induced with 0.2 mM IPTG at 16 °C. Bacteria expressing GST-tagged protein were suspended and lysed by sonication in PBS buffer supplemented with 0.1% Triton X-100. The preparation was incubated with glutathione-Sepharose 4B (GE Healthcare Life Science) for 1.5 h at 4 °C. The resin was washed three times, and GST-tagged protein was eluted with 10 mM reduced glutathione. Bacteria expressing MBP-AMPKα2 were lysed in MBP column buffer (20 mM Tris-HCl, pH 7.4; 200 mM NaCl; 1 mM EDTA) and incubated with amylose resin (New England BioLabs) for 1.5 h at 4 °C. The resin was washed three times in MBP column buffer and eluted with MBP column buffer supplemented with 10 mM maltose. All purification procedures were performed at 4 °C, and protease inhibitor cocktail (Sigma) was added to prevent protein degradation.

### Immunoprecipitation and pull-down assays

For immunoprecipitation, cells were treated with indicated reagents before being lysed in lysis buffer (50 mM Tris-HCl, pH 7.5; 120 mM NaCl; 0.2% NP-40; 1 mM EDTA; 1 mM DTT) supplemented with protease inhibitor cocktail (Sigma), phosphatase inhibitor cocktail (Sigma). After preclearing with protein A/G resin, the lysate was incubated with indicated antibody at 4 °C for 24 h with gentle rotation. Protein A/G resin was then added to the lysates, and they were incubated for another 4 h. The protein A/G resin was then spun down and washed four times with lysis buffer before being resolved by SDS-PAGE and immunoblotted with the indicated antibodies. For FLAG-tagged protein immunoprecipitation, the FLAG-M2 resin was added to the lysates and incubated for 4 h before washing. For *in vitro* reactions, the FLAG beads were further washed twice with dialysis buffer, and the FLAG-tagged protein was eluted with dialysis buffer supplemented with 100 μg/ml 3×FLAG peptide (Sigma). For pull-down assays, GST or GST-tagged proteins were purified from bacteria and incubated with cell lysates or eluted His-tagged protein for 4 h. Four hours later, the bound fraction was washed with lysis buffer four times and analyzed by Western blotting.

### Western blot

Whole-cell extracts were prepared with SDS-PAGE sample buffer. Proteins were separated in SDS-PAGE with 10% gels and transferred onto nitrocellulose membranes (Millipore). Uniform transfer was confirmed by Ponceau S staining. Membranes were blocked in Tris-buffered saline with 0.1% Tween 20 (TBS-T buffer) and 5% dry milk (w/v) for 1 h at room temperature and washed three times in TBS-T. Membranes were blotted overnight at 4 °C with primary antibodies diluted at an appropriate dilution ratio in TBS-T with 5% dry milk and then with a secondary donkey anti-rabbit IgG antibody conjugated with horseradish peroxidase (Cell Signaling Technology) for 1 h at room temperature. After washing, signals were developed with the ECL detection system (Thermo Scientific).

### *In vitro* kinase assay

The kinase reactions were performed in 40 μl kinase buffer (25 mM Hepes, pH 7.4; 100 mM NaCl; 5 mM MgCl_2_; 1 mM DTT) with purified Sirt1 (2 μg) substrate, LKB1 kinase (100 ng His-LKB1-STRADα-MO25α tri-complex, expressed in baculovirus system), 50 μM ATP with or without 5 μCi of [γ-^32^P]-ATP. Reaction mixtures were incubated at 30 °C for 30 min, then terminated by 5× SDS-PAGE loading buffer (10% SDS, 0.5% bromophenol blue, 50% glycerol, 100 mM DTT). After the samples were boiled at 100 °C for 2 min, 50% of the sample was resolved by SDS-PAGE. For the reaction without [γ-^32^P]-ATP, samples were subjected to Western blotting with indicated antibodies to test phosphorylation levels. For the reaction in the presence of [γ-^32^P]-ATP, samples were stained by Coomassie Brilliant Blue R250 (CBB). For autoradiograms, CBB-stained SDS-PAGE gels were destained, scanned, and then dried between sheets of cellulose for 4 h. The semidried gels were then placed between intensifier and X-ray films for 12 to 24 h in a −80 °C freezer, and the levels of ^32^P incorporation into Sirt1 proteins was tested.

### Fluorometric Sirt1 activity assay

FLAG-Sirt1 was immunoprecipitated from cells infected with pLVX-FLAG-Sirt1 lentivirus particles and was eluted by FLAG peptide. Then 50 ng eluted FLAG-Sirt1 was incubated with 1 mM of NAD^+^ and 200 μM fluorescently labeled acetylated p53 peptide (Enzo Life Sciences) in Sirt1 assay buffer (50 mM Tris-HCl, pH 8.0; 137 mM NaCl; 2.7 mM KCl; 1 mM MgCl_2_; 1 mg/ml BSA). The plate was incubated at 37 °C for 30 min, and the reaction was stopped with developer solution (Enzo Life Sciences) containing 2 mM nicotinamide to inhibit Sirt1 and protease to digest deacetylated p53 peptide. Sirt1 activity was assessed by measuring the fluorescent emission at 460 nm, following excitation at 360 nm.

### LKB1 kinetics assay

The kinetics of LKB1 was characterized by the AmpliteTM Universal Fluorometric Kinase Assay Kit (AAT Bioquest) according to the manufacturer’s instructions. In brief, 200 nM recombinant LKB1 kinase was incubated with gradient concentrations of purified Sirt1 protein in 20 μl kinase reaction buffer (ADP assay buffer) at 37 °C for 30 min; 20 μl ADP sensor buffer and 10 μl ADP sensor were added into the preparations to make a total 50 μl ADP assay volume. The reaction mixture was incubated at room temperature for 30 min. The amount of ADP produced from the kinase reaction assay was detected by monitoring the fluorescence intensity at Ex/Em = 540/590 nm. The background fluorescence was determined by measuring the fluorescence intensity in the absence of substrate and subtracted from the experiments. *K*_*m*_ and *k*_cat_ values were calculated by the Michaelis–Menten equation as reported (83).

### LC-MS analysis

To prepare samples for mass spectrometric analysis of phosphorylation site(s) of Sirt1 by LKB1, Sirt1 protein purified from *E. coli* was incubated with recombinant LKB1-STRADα-Mo25α complex in the presence of ATP and then separated by SDS-PAGE and depicted with colloidal Coomassie blue staining. Following reduction with dithiothreitol and alkylation with iodoacetamide, in-gel digestion of Sirt1 was performed with sequencing-grade Glu-C (Promega, V1651) at 1/50 (Glu-C/protein, w/w) ratio at 37 °C for 16 h in 50 mM NH_4_HCO_3_ (pH 8.0). The samples were then dried and dissolved in 0.1% formic acid (FA) water. LC-MS analysis was performed on ACQUITY UPLC M-Class (Waters) and XEVO G2-XS QTof Mass Spectrometer System (Waters). The Sirt1 protein samples were separated by a 120-min gradient with the linear gradient from 3% to 80% B (A = 0.1% FA in H_2_O, B = 0.1% FA in acetonitrile) at a flow rate of 300 nl/min and the Sirt1-669-peptide samples were separated by a 30-min gradient with the linear gradient from 3% to 80% B (A = 0.1% FA in H_2_O, B = 0.1% FA in acetonitrile) at a flow rate of 300 nl/min. The QToF mass spectrometer was operating in MSE mode or target MSMS mode, and the raw data were searched with Progenesis QI for proteomics V4.1 Software against the Human uniprot database.

### Immunofluorescence

C2C12, HEK293T, or HeLa cells grown on coverslips were fixed by a pre-extraction method using PTEM buffer (60 mM Pipes, pH 6.8; 10 mM EGTA; 2 mM MgCl_2_; 0.2% Triton X-100) supplemented with 3.7% paraformaldehyde. After blocking with PBST (PBS with 0.05% Tween-20) buffer containing 1% bovine serum albumin (Sigma) for 45 min at room temperature, the fixed cells were incubated with primary antibodies in a humidified chamber for 1 h at room temperature or overnight at 4 °C, followed by secondary antibodies for 1 h at 37 °C. The DNA was stained with DAPI from Sigma. Images were captured by Delta Vision softWoRx software (Applied Precision) and processed by deconvolution and z-stack projection.

### Gene expression and mtDNA analysis

RNA from C2C12 cells were extracted with E.Z.N.A. Total RNA Kit (OMEGA) according to the instructions and quantified using the NanoDrop 1000 spectrophotometer (Thermo Scientific). The cDNA was synthesized with the HiScript Ⅲ RT SuperMix for qPCR (Vazyme) using 1 μg of RNA. Quantitative RT-PCR reactions were performed using 1 μM of primers and LightCycler 480 SYBR Green Master (Roche) on a LightCycler 480 detection system (Roche). Calculations were performed by a comparative method (2-ΔΔCT) using GAPDH as an internal control. Primers used for PCR analysis are listed:

GAPDH:

Forward primer: 5′-ACATCATCCCTGCATCCACTG-3′

Reverse primer: 5′-CCTGCTTCACCACCTTCTTG-3′

Sirt1:

Forward primer: 5′-GGCCGCGGATAGGTCCATA-3′

Reverse primer: 5′-ACAATCTGCCACAGCGTCAT-3′

LKB1:

Forward primer: 5′-CTGGACTCCGAGACCTTATGC-3′

Reverse primer: 5′-CAAGCTGGATCACATTCCGAT-3′

PGC-1α:

Forward primer: 5′-TATGGAGTGACATAGAGTGTGCT-3′

Reverse primer: 5′-GTCGCTACACCACTTCAATCC-3′

NRF1:

Forward primer: 5′-AATGACCCAGGCTCAGCTTC-3′

Reverse primer: 5′-GCTTGCAGCTTTCTTTCCCC-3′

NRF2:

Forward primer: 5′-TGACCATGAGTCGCTTGCC-3′

Reverse primer: 5′-TCCTGCCAAACTTGCTCCAT-3′

NDUFS8:

Forward primer: 5′-GTTCATAGGGTCAGAGGTCAAG-3′

Reverse primer: 5′-TCCATTAAGATGTCCTGTGCG-3′

SDHb:

Forward primer: 5′-GAGTCGGCCTGCAGTTTCA-3′

Reverse primer: 5′-GGTCCCATCGGTAAATGGCA-3′

Uqcrc1:

Forward primer: 5′-GTGTCTCATTTGGATGGCACC-3′

Reverse primer: 5′-AGCAAATGTCACGCAGCATC-3′

Cox5b:

Forward primer: 5′-GCTTCAAGGTTACTTCGCGG-3′

Reverse primer: 5′-ATGGGTCCAGTCCCTTCTGT-3′

ATP5a1:

Forward primer: 5′-CATTGGTGATGGTATTGCGC-3′

Reverse primer: 5′-TCCCAAACACGACAACTCC-3′

For mtDNA analysis, total DNA was extracted with E.Z.N.A. Total DNA Kit (OMEGA). The mtDNA was amplified using primers specific for the mitochondrial cytochrome c oxidase subunit 2 (COX2) gene and normalized to genomic DNA by amplification of the ribosomal protein s18 (rps18) nuclear gene. Primers were designed using the IDT software (IDT) and the primer sequences are listed:

COX2:

Forward primer: 5′-ATAACCGAGTCGTTCTGCCAAT-3′

Reverse primer: 5′-TTTCAGAGCATTGGCCATAGAA-3′

Rps18:

Forward primer: 5′-TGTGTTAGGGGACTGGTGGACA-3′

Reverse primer: 5′-CATCACCCACTTACCCCCAAAA-3′

### Cell fractionation

Cells were washed twice with iced PBS and collected into hypotonic buffer (10 mM Hepes, pH 8.0, 10 mM KCl, 3 mM MgCl_2_, 0.5 mM DTT, 0.2% Triton X-100, and protease inhibitors). Cell lysates were incubated on ice for 10 min and then centrifuged at 500*g* for 5 min at 4 °C; the supernatant was used as the cytoplasmic fraction. The pellet was washed twice with hypotonic buffer and resuspended in RIPA buffer (100 mM Tris-HCl, pH 8.0, 1% Triton X-100, 100 mM NaCl, 0.5 mM EDTA, and protease inhibitors). After centrifugation at 15,000*g* for 10 min, the supernatant was used as the nuclear fraction (84).

### Mitochondrial density and mitochondrial membrane potential measurements by flow cytometry

For measuring mitochondrial density, the cells were resuspended in PBS containing 50 nM MitoTracker Red CMXRos (Invitrogen), and incubated for 30 min at 37 °C according to the manufacturer’s instructions. The fluorescence was excited at 561 nm in a flow cytometer (Beckman, Moflo Astrios TM). Data were analyzed using FlowJo software for 2 × 10^4^ cells as described (85).

For measuring mitochondrial membrane potential, the cells were resuspended in PBS containing 20 nM tetramethyl rhodamine methyl ester (Invitrogen) and incubated for 30 min at 37 °C according to the manufacturer’s instructions. The fluorescence was excited at 561 nm in a flow cytometer (Amnis, ImageStream mark ii). Data were analyzed using IDEAS 6.2 software for 2 × 10^3^ cells.

### Oxygen consumption rate measurements in C2C12 myotubes

C2C12 cells were seeded in XF 96-well cell culture microplates (Seahorse biosciences) at 1 × 10^4^ cells/well in 80 μl high-glucose DMEM growth medium supplemented with 10% fetal bovine serum and 1% pen/strep. The following day, cells were infected with virus for 12 h and continuously cultured in fresh medium for 36 h. Then, cells were switched to low-serum media containing 2% horse serum and 1% pen/strep to induce differentiation. Cells were fed every 24 h for 4 days. On the day of testing, 25 μM resveratrol or DMSO as a vehicle control was suspended in fresh DMEM media and the cells were returned to the incubator for 6 h. After 6 h, cells were washed twice with 120 μl/well assay medium. A final volume of 180 μl of assay medium was added to each well, and cells were then transferred to a CO_2_-free incubator, maintained at 37 °C for 1 h before the start of the assay. Following assay calibration, measurements of oxygen consumption rate were performed with an XF96 Extracellular Flux Analyzer (Seahorse biosciences) as per the manufacturer’s protocol.

### Statistics

Statistical analyses were performed using GraphPad Prism 7.0. Statistical differences were determined by unpaired two-tailed Student’s *t* test between two groups, one-way ANOVA with Tukey’s multiple comparison tests as a post hoc test for comparing every mean to every other mean, or one-way ANOVA with Dunnett’s multiple comparison as a post hoc test for comparing every mean to a control mean in each data set. A *p*-value <0.05 was considered statistically significant. Data are presented as means ± SD. Statistical tests used are indicated in the corresponding figure legends.

## Data availability

All data are contained in the article. Raw mass spectrometry data are deposited in a publicly accessible repository Zenodo. The DOI is 10.5281/zenodo.4775266.

## Supporting information

This article contains supporting information.

## Conflict of interest

The authors declare that they have no conflicts of interest with the contents of this article.
