# Supplementary material for: Resveratrol-induced Sirt1 phosphorylation by LKB1 mediates mitochondrial metabolism
Source: J Biol Chem. 2021 Jul 1;297(2):100929. doi: 10.1016/j.jbc.2021.100929 (PMC8326426; doi:10.1016/j.jbc.2021.100929)
Supplement: Supplemental Figures S1–S5 [file mmc1.pdf]

Supporting Information

Resveratrol-induced Sirt1 phosphorylation by LKB1 mediates mitochondrial metabolism

Yuanyuan Huang[1,2,4], Jianlin Lu[1,2,4], Li Zhan[1,2], Ming Wang[1,2], Ronghua Shi[1,2], Xiao Yuan[1,2], Xinjiao Gao[1,2], Xing Liu[1,2], Jianye Zang[1,2], Wei Liu[3], and Xuebiao Yao[1,2]

[1]MOE Key Laboratory for Membraneless Organelles and Cellular Dynamics, University of Science & Technology of China School of Life Sciences, Hefei 230027, China; [2]Anhui Key Laboratory for Cellular Dynamics & Chemical Biology, CAS Center for Excellence in Molecular Cell Science & Hefei National Science Center for Physical Sciences at Microscale, Hefei, Anhui 230026, China; [3]Zhejiang University School of Medicine, Hangzhou, 310058, Zhejiang, China

[4]These two authors contributed equally

Running title: *LKB1-Sirt1 signaling in mitochondrial metabolism*

*Correspondence: yaoxb@ustc.edu.cn

Keywords: mitochondria, phosphorylation, LKB1, Sirt1, deacetylation

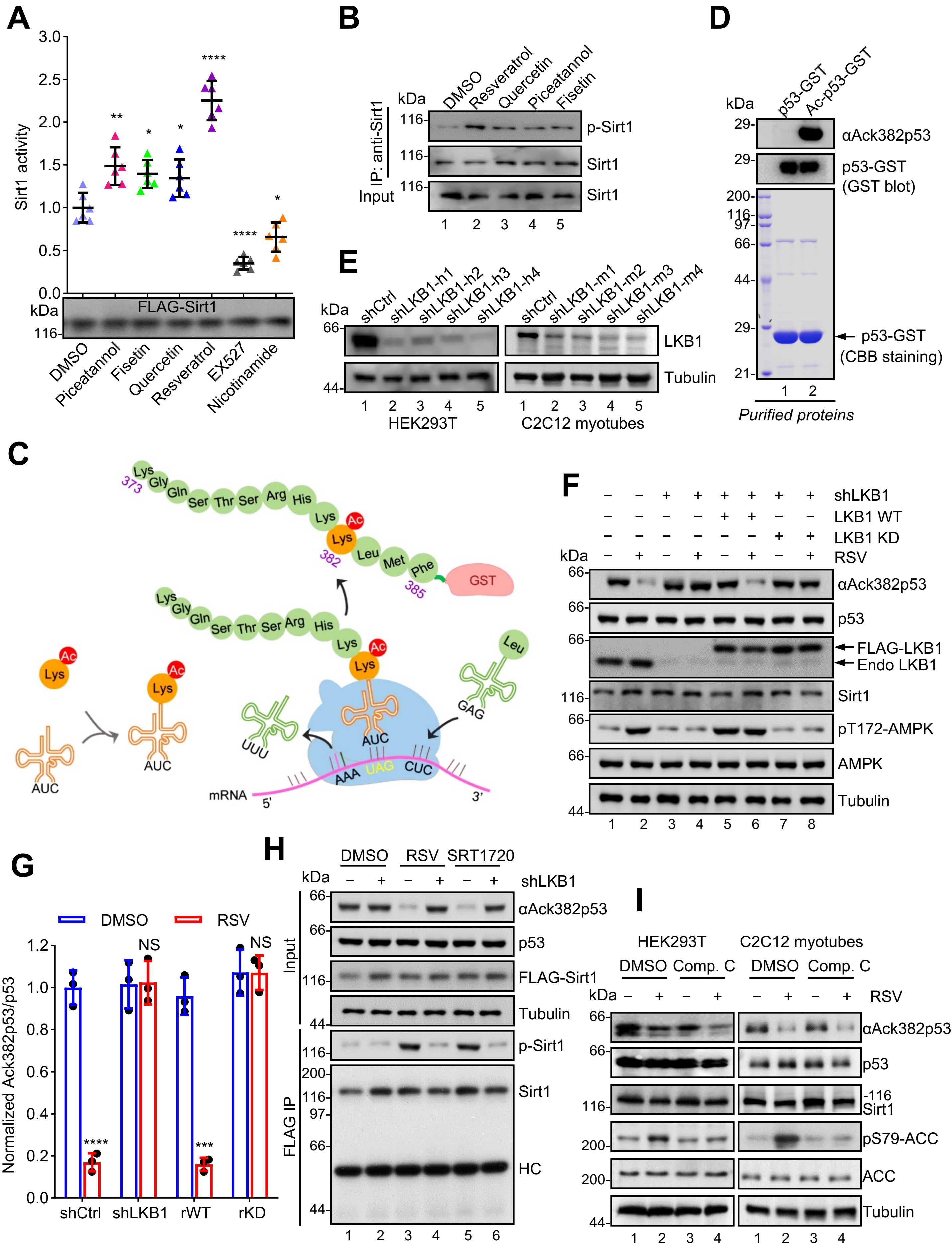
A
Sirt1 activity
3.0
2.5
2.0
1.5
1.0
0.5
0.0
**
*
*
****
****
*
kDa
FLAG-Sirt1
116-
DMSO
Piceatannol
Fisetin
Quercetin
Resveratrol
EX527
Nicotinamide
B
kDa
DMSO
Resveratrol
Quercetin
Piceatannol
Fisetin
IP: anti-Sirt1
116-
p-Sirt1
116-
Sirt1
Input
116-
Sirt1
1 2 3 4 5
D
kDa
p53-GST
Ac-p53-GST
29-
αAck382p53
29-
p53-GST
(GST blot)
200-
116-
97-
66-
44-
29-
21-
p53-GST
(CBB staining)
1 2
Purified proteins
E
kDa
shCtrl
shLKB1-h1
shLKB1-h2
shLKB1-h3
shLKB1-h4
shCtrl
shLKB1-m1
shLKB1-m2
shLKB1-m3
shLKB1-m4
66-
LKB1
44-
Tubulin
1 2 3 4 5
HEK293T
1 2 3 4 5
C2C12 myotubes
C
Lys Gly Gln Ser Thr Ser Arg His Lys Lys Leu Met Phe
373
382
385
Ac
GST
Lys
Ac
AUC
UUU
AAA UAG CUC
GAG
Leu
mRNA
5'
3'
F
shLKB1
LKB1 WT
LKB1 KD
kDa
RSV
66-
αAck382p53
66-
p53
66-
FLAG-LKB1
Endo LKB1
116-
Sirt1
66-
pT172-AMPK
66-
AMPK
44-
Tubulin
1 2 3 4 5 6 7 8
G
DMSO
RSV
Normalized Ack382p53/p53
1.2
1.0
0.8
0.6
0.4
0.2
0.0
NS
NS
****
***
shCtrl
shLKB1
rWT
rKD
H
DMSO
RSV
SRT1720
kDa
shLKB1
Input
66-
αAck382p53
66-
p53
116-
FLAG-Sirt1
44-
Tubulin
FLAG IP
116-
p-Sirt1
200-
116-
97-
66-
44-
Sirt1
HC
1 2 3 4 5 6
I
HEK293T
C2C12 myotubes
DMSO
Comp. C
DMSO
Comp. C
kDa
RSV
66-
αAck382p53
66-
p53
116-
-116
Sirt1
200-
pS79-ACC
200-
ACC
44-
Tubulin
1 2 3 4
1 2 3 4

Figure S1. LKB1-dependent phosphorylation is required for resveratrol-induced Sirt1 activation. *A*, The activity of Sirt1 that were quantified by using a fluorophore-conjugated acetylated p53 peptide. C2C12 myoblasts were infected with lentivirus-based particles expressing FLAG-Sirt1 and then were cultured with 2% horse serum for 4 days to generate myotubes. Cells were treated with Sirt1 activators for 6 hours, including 25 μM piceatannol, 25 μM fisetin, 25 μM quercetin or 25 μM resveratrol. Sirt1 inhibitors (1 μM EX527 or 10 mM nicotinamide) treatment were shown as negative controls. Data represents mean ± SD. The experiment was repeated six times independently. Statistical significance was determined by Dunnett's multiple comparisons test. **p (piceatannol) =0.0014, *p (fisetin) =0.0117, *p (quercetin) =0.0323, ****p (resveratrol) <0.0001, ****p (EX527) <0.0001, *p (nicotinamide) =0.0336. *B*, Phosphorylation of Sirt1 in C2C12 myotubes treated with Sirt1 activators as indicated in Fig. S1A. Sirt1 proteins were immunoprecipitated by anti-Sirt1 and immunoblotted with anti-phospho-serine/threonine. Representative of three independent experiments. *C*, Diagram of plasmid construction for producing recombinant acK382-p53 peptide in *E. coli* by using non-natural amino acid technology. *D*, Purified wild-type p53 peptide and p53 peptide bearing Lys382 acetylation were analyzed by using anti-acetyl-p53 K382 antibody. The p53 peptide, aa 373-385. The acK382, deacetylation site of Sirt1. *E*, Knockdown efficiency of lentivirus-based LKB1 shRNAs analyzed by Western blotting in HEK293T and C2C12 myotubes. The h1-h4, four shRNAs for HEK293T cells. The m1-m4, four shRNAs for C2C12 myotubes. The selected LKB1 shRNA used in HEK293T was shLKB1-h4 and shLKB1-m4 was used in C2C12 myotubes. *F*, The deacetylase activity of Sirt1 in resveratrol-treated LKB1-depleted HEK293T cells expressing WT LKB1 or kinase-dead mutant. The experimental replicate of Fig. 1F. *G*, Quantification of Ack382p53 as in Fig. 1F. Data represent mean ± SD. The experiment was repeated three times independently. ****p<0.0001. ***p=0.0001. NS (not significant) indicates p>0.05. Unpaired two-tailed *t*-test for DMSO treatment and resveratrol treatment pair in each group. *H*, The deacetylase activity of Sirt1 in SRT1720-treated LKB1-depleted cells. HEK293T cells were infected with lentivirus-based particles expressing shRNA control or LKB1 shRNA for 12 hours. After 48 hours, cells were pretreated with 1 μM doxorubicin for 1 hour to increase in vivo K382 acetylation of p53 (deacetylation site of Sirt1) and then were treated with 25 μM resveratrol or 1 μM SRT1720 for 6 hours. The whole-cell lysate (WCL) were immunoblotted with anti-acetyl-p53 K382. Representative of three independent experiments. *I*, The activity of Sirt1 in cells treated with AMPK inhibitor. Cells were pretreated with 1 μM doxorubicin for 1 hour and then were treated with 10 μM Compound C (AMPK inhibitor) and 25 μM resveratrol for 6 hours. WCL were immunoblotted with anti-acetyl-p53 K382. Representative of three independent experiments. The pS79-ACC, marker of AMPK activity. The p53 K382 is the deacetylation site of Sirt1 and ack382-p53 is the marker of Sirt1 activity.

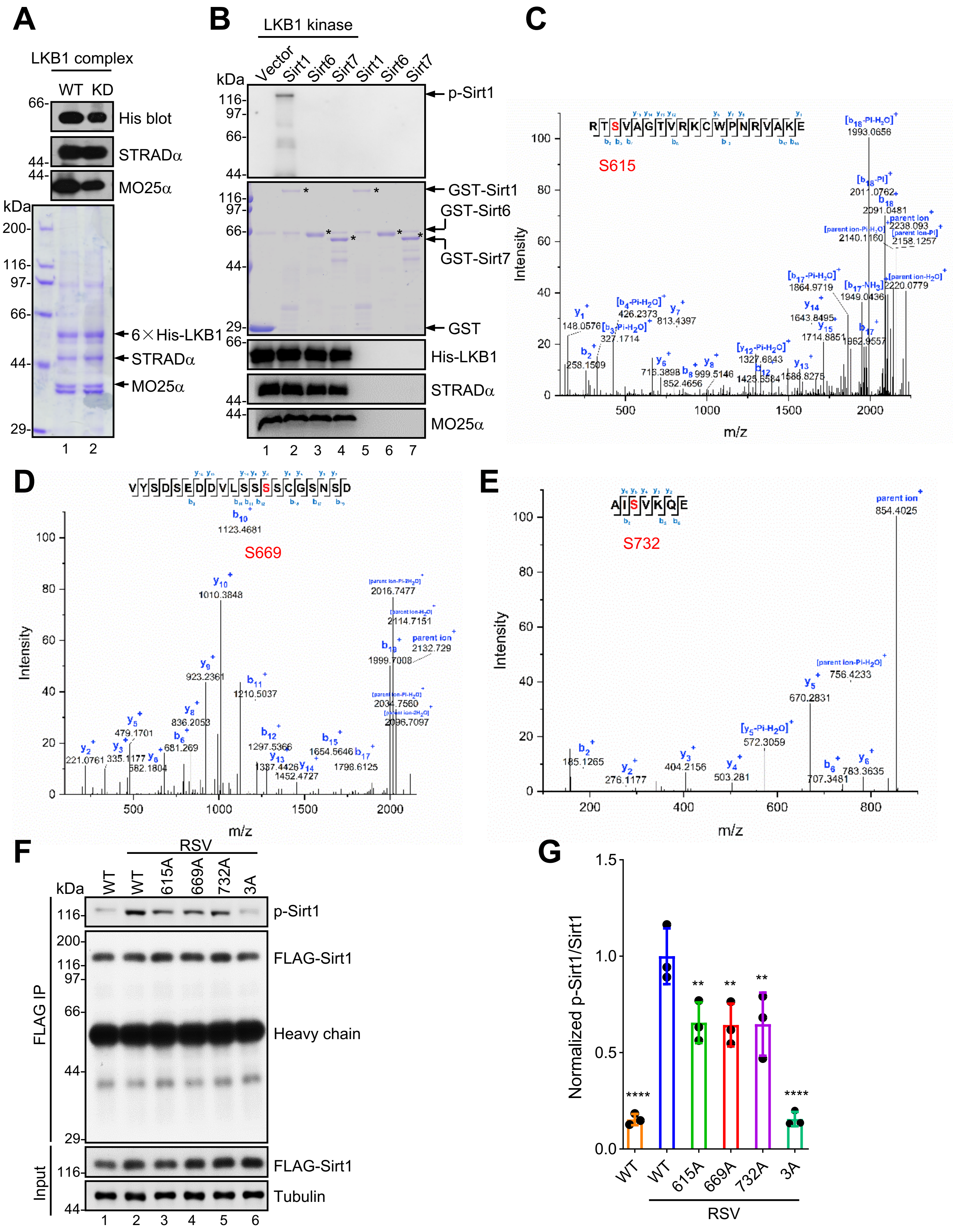
A
LKB1 complex
WT KD
His blot
STRADα
MO25α
kDa
6×His-LKB1
STRADα
MO25α
1 2
B
LKB1 kinase
kDa
Vector Sirt1 Sirt6 Sirt7 Sirt1 Sirt6 Sirt7
p-Sirt1
GST-Sirt1
GST-Sirt6
GST-Sirt7
GST
His-LKB1
STRADα
MO25α
1 2 3 4 5 6 7
C
RTSVAGTVRKCWPNRVAKE
S615
Intensity
m/z
D
VYSDSEDDVLSSSSCGSNSD
S669
Intensity
m/z
E
AISVKQE
S732
Intensity
m/z
F
RSV
kDa
WT WT 615A 669A 732A 3A
p-Sirt1
FLAG-Sirt1
Heavy chain
FLAG-Sirt1
Tubulin
FLAG IP
Input
1 2 3 4 5 6
G
Normalized p-Sirt1/Sirt1
WT WT 615A 669A 732A 3A
RSV

Figure S2. Sirt1 is directly phosphorylated by LKB1. *A*, Recombinant 6×His-LKB1-STRADα-MO25α complex (LKB1 kinase) purified from baculovirus-infected insect cells were analyzed by Western blotting and CBB staining as indicated. WT, wild-type. KD, kinase-dead, Lys78Met. *B*, *In vitro* kinase assay using recombinant LKB1 kinase and purified GST-Sirt1, GST-Sirt6, or GST-Sirt7. Samples were subjected to immunoblotting as indicated. Representative of three independent experiments. *C*-*E*, Mass spectrometry of Sirt1 phosphorylation. To prepare samples for mass spectrometric analysis of phosphorylation site(s) of Sirt1 by LKB1, recombinant Sirt1 was incubated with recombinant LKB1-STRADα-Mo25α complex in the presence of ATP, and then separated by SDS-PAGE and depicted with colloidal Coomassie blue staining. Following reduction and alkylation, in-gel digestion of Sirt1 was performed with protease Glu-C (Promega, V1651), a serine protease that specifically cleaves at the C-terminus of either aspartic or glutamic acid residues. The 0.1% formic acid was added to the mobile phase to enhance the ionization efficiency in the positive ion mode. *F*, Phosphorylation levels of Sirt1 in HEK293T cells expressing FLAG-Sirt1 wild-type, FLAG-Sirt1 S615A, FLAG-Sirt1 S669A, FLAG-Sirt1 S732A, or FLAG-Sirt1 3A. The experimental replicate of Fig. 2D. *G*, Quantification of phospho-Sirt1 as in Fig. 2D. Data represent mean ± SD. The experiment was repeated three times independently. Statistical significance was determined by Dunnett's multiple comparisons test. ****$p<0.0001$. **p (S615A) =0.0099, **p (S669A) =0.0078. **p (S732A) =0.0085.

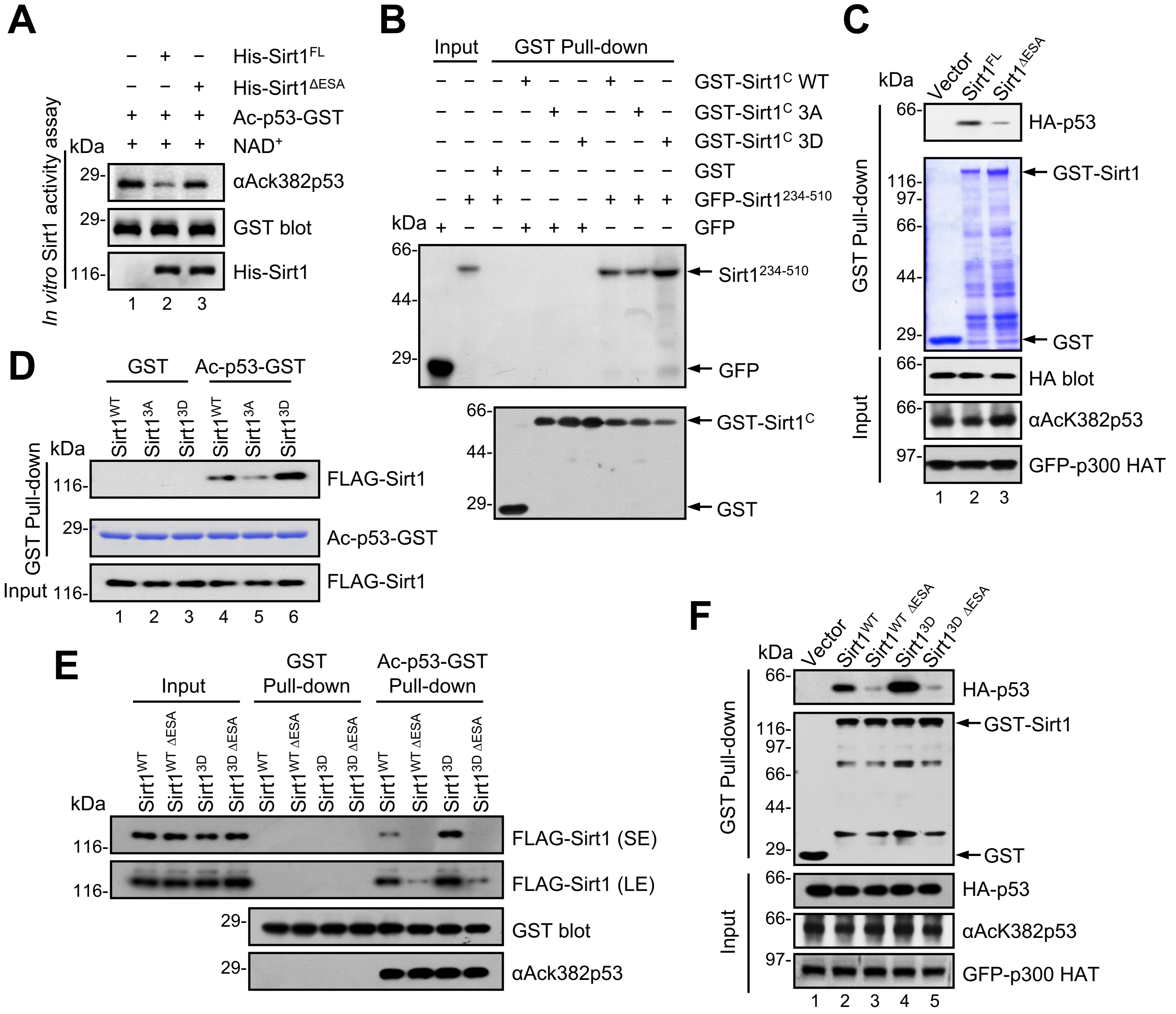

Figure S3. Phosphorylation by LKB1 promotes the C-terminus-Deacetylase core interaction and Sirt1-Substrate interaction. *A*, *In vitro* Sirt1 deacetylase activity assay. Baculovirus expressed His-tagged Sirt1 deacetylase (aa 193-747) (FL, full length, or the ΔESA mutant) was incubated with GST-tagged K382ac p53 peptide. Acetylation level of K382 site was analyzed by using anti-acetyl-p53 K382 antibody. ΔESA, ESA deletion. The p53 K382 is the deacetylation site of Sirt1 and ack382-p53 is the marker of Sirt1 activity. *B*, GST pull-down assay using GST-tagged C-terminus of Sirt1 (WT, or the mutants) proteins and lysates of HEK293T cells transfected with GFP-tagged core domain of Sirt1. Sirt1^C, C-terminus. 3A, Ser615, Ser669, and Ser732 were replaced by Ala. 3D, Ser615, Ser669, and Ser732 were replaced by Asp. *C*, GST pull-down assay using recombinant GST-Sirt1 (FL, full length, or the ΔESA mutant) and lysates of HEK293T cells transfected with HA-tagged p53 and GFP-tagged p300 HAT domain. ΔESA, ESA deletion. HAT domain, histone acetyltransferase domain of p300, aa 3583-5280. Ack382-p53 was used as the substrate of Sirt1. *D*, GST pull-down assay using recombinant GST-tagged K382ac p53 peptide (aa 373-385) and lysates of HEK293T cells transfected with FLAG-Sirt1 (WT, or the mutants). 3A, Ser615, Ser669, and Ser732 were replaced by Ala. 3D, Ser615, Ser669, and Ser732 were replaced by Asp. Ack382-p53 was used as the substrate of Sirt1. *E*, GST pull-down assay using recombinant GST-tagged K382ac p53 peptide and lysates of HEK293T cells transfected with FLAG-Sirt1 (WT, or the mutants). SE, short exposure. LE, longer exposure. Ack382-p53 was used as the substrate of Sirt1. *F*, GST pull-down assay using recombinant GST-Sirt1 (WT, or the mutants) and lysates of HEK293T cells transfected with HA-tagged p53 and GFP-tagged p300 HAT domain. ΔESA, ESA deletion. HAT domain, histone acetyltransferase domain of p300, aa 3583-5280. Ack382-p53 was used as the substrate of Sirt1.

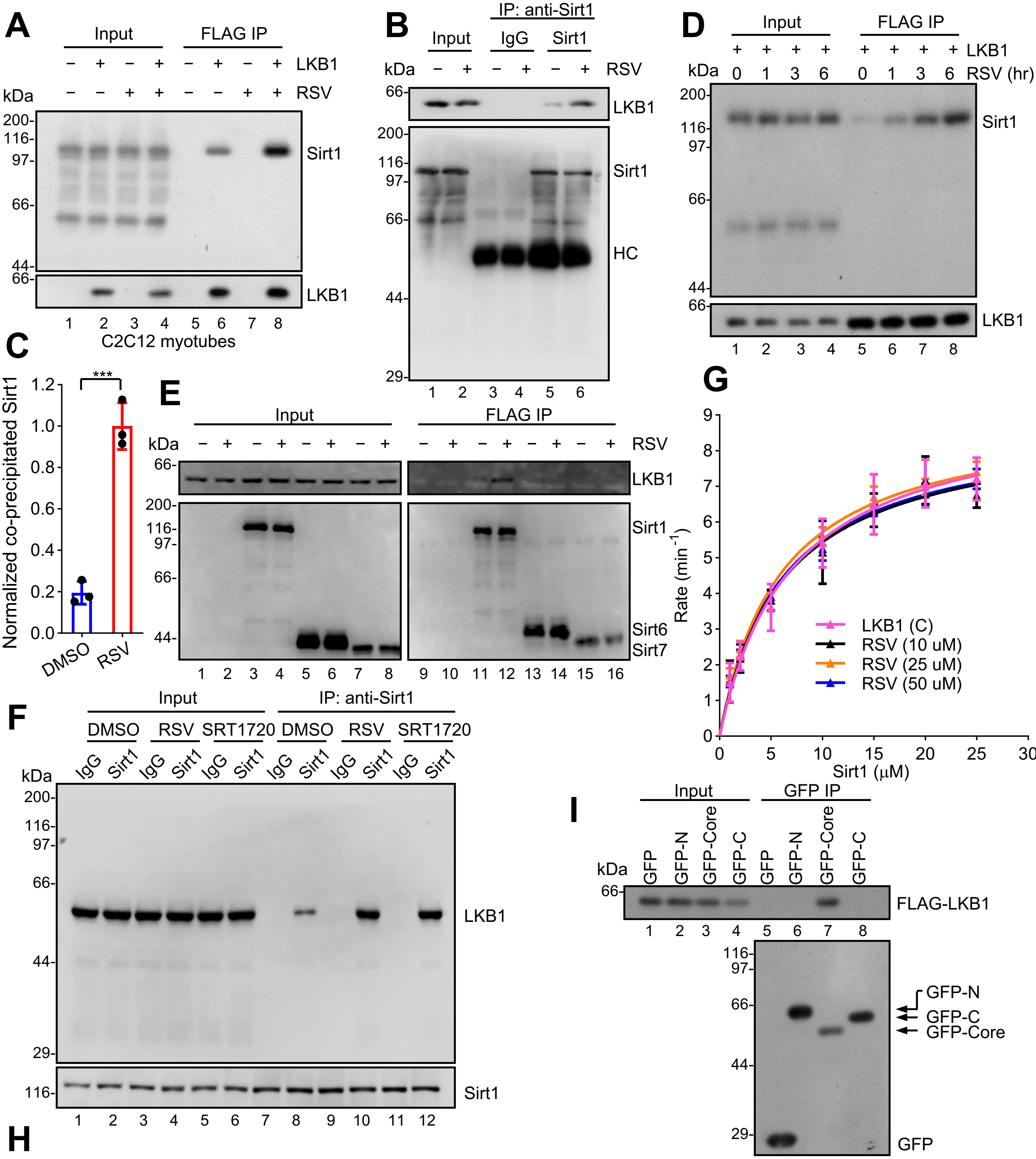

H

	LKB1 (C)	RSV (10 μM)	RSV (25 μM)	RSV (50 μM)
K_m (μM)	6.928±0.8579	6.157±0.7073	5.950±0.8048	6.030±0.7274
k_{cat} (min^{-1})	9.331±0.4043	8.782±0.3360	9.111±0.4046	8.812±0.3510
k_{cat}/K_m (μM^{-1}·min^{-1})	1.347	1.426	1.531	1.461

Figure S4. Resveratrol promotes the binding affinity of LKB1 and Sirt1. *A*, Immunoprecipitation of FLAG-LKB1 with Sirt1 in resveratrol-treated C2C12 myotubes. Cells were infected with lentivirus-based particles expressing FLAG-LKB1 and then were treated with 25 μM resveratrol (RSV) for 6 hours. FLAG-LKB1 proteins were immunoprecipitated by anti-FLAG and the bound fraction was analyzed by using anti-Sirt1 antibody. The experiment was repeated three times independently. *B*, Immunoprecipitation of Sirt1 with endogenous LKB1 in resveratrol-treated C2C12 myotubes. The experimental replicate of Fig. 4C. *C*, Quantification of precipitated Sirt1 as in Fig. 4C. Data represent mean ± SD. The experiment was repeated three times independently. ***p=0.0004. Unpaired two-tailed *t*-test for DMSO treatment and resveratrol treatment. *D,* Immunoprecipitation of FLAG-LKB1 with Sirt1 in resveratrol-treated HEK293T cells. HEK293T cells were infected with lentivirus-based particles expressing FLAG-LKB1 and then were treated with 25 μM resveratrol (RSV) for 0 hour, 1 hour, 3 hours, or 6 hours. FLAG-LKB1 proteins were immunoprecipitated by anti-FLAG and the bound fraction was analyzed by using anti-Sirt1 antibody. The experiment was repeated three times independently. *E*, Immunoprecipitation of FLAG-Sirt1, FLAG-Sirt6, or FLAG-Sirt7 with LKB1 in resveratrol-treated HEK293T cells. Cells were transfected with FLAG-Sirt1, FLAG-Sirt6, or FLAG-Sirt7 and then were treated with 25 μM resveratrol (RSV) for 6 hours. FLAG-tagged proteins were immunoprecipitated by anti-FLAG and the bound fraction was analyzed by using anti-LKB1 antibody. The experiment was repeated three times independently. *F*, Immunoprecipitation of Sirt1 with LKB1 in SRT1720-treated C2C12 myotubes. C2C12 myoblast cells were grown in DMEM with 2% horse serum for 4 days. Cells were treated with 1 μM SRT1720 or 25 μM resveratrol for 6 hours. Lysates were subjected to immunoprecipitation using antibody against Sirt1. The precipitates were examined by immunoblotting with anti-LKB1 antibody. The experiment was repeated three times independently. *G*, Kinetics curves of LKB1 kinase were measured at various concentrations of Sirt1 protein in the indicated conditions. The background fluorescence was determined by measuring the fluorescence intensity in the absence of substrate and subtracted from the experiments. Data represent mean ± SD. LKB1 (C), the kinetics assay was added with DMSO. RSV, resveratrol. *H*, The enzyme kinetics were calculated according to the Michaelis-Menten equation in the conditions indicated, related to Fig. S4G. *I*, Co-immunoprecipitation of FLAG-LKB1 with GFP-tagged fragments of Sirt1. HEK293T cells were transiently transfected with FLAG-LKB1 and GFP-tagged Sirt1's N-terminus (aa 1-233), deacetylase core (aa 234-510), or C-terminus (aa 511-747). Then the cell lysates were immunoprecipitated with anti-GFP antibody, and the immunocomplexes were subjected to western blotting with anti-FLAG antibody. The experiment was repeated three times independently.

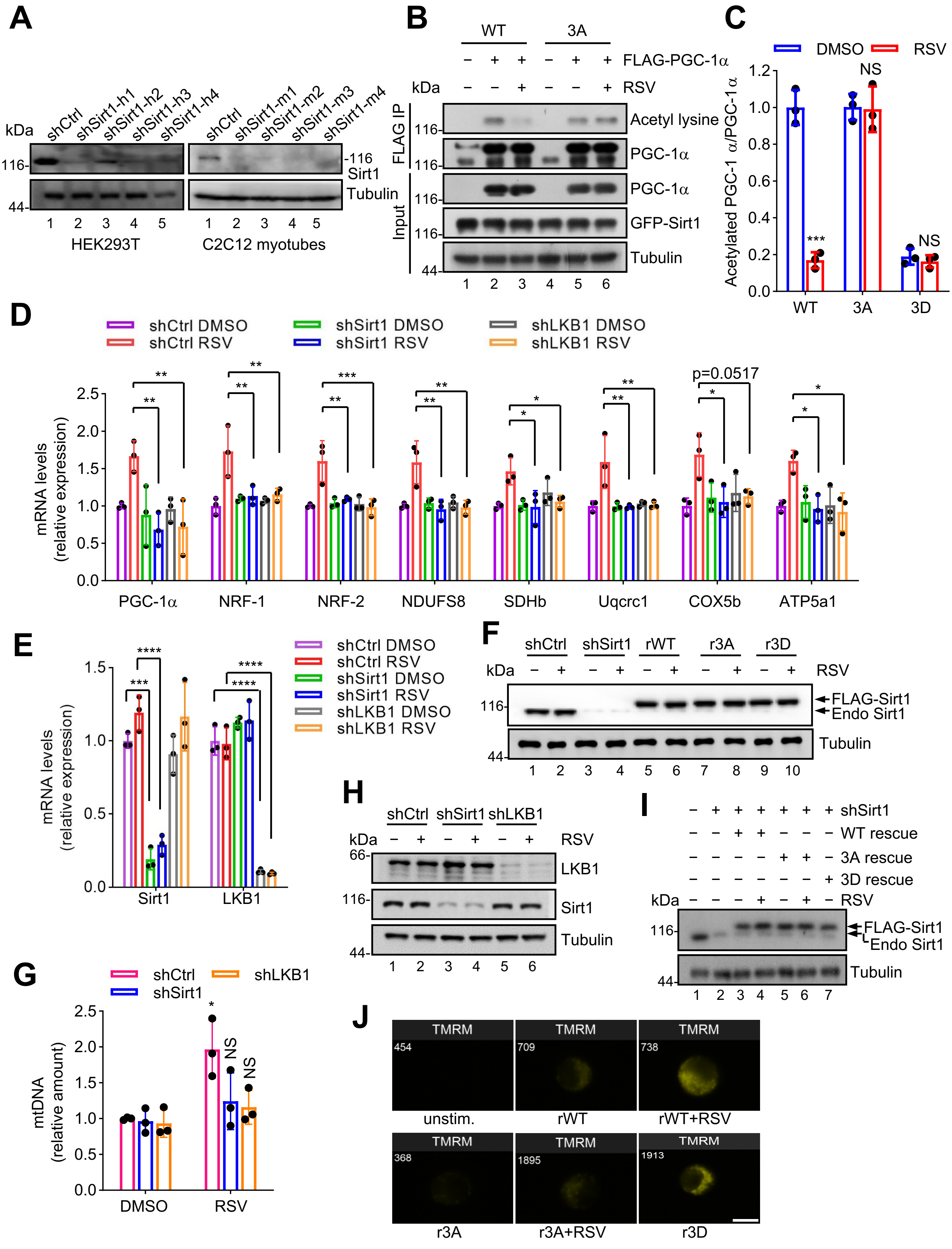
A
kDa
shCtrl
shSirt1-h1
shSirt1-h2
shSirt1-h3
shSirt1-h4
shCtrl
shSirt1-m1
shSirt1-m2
shSirt1-m3
shSirt1-m4
116-
44-
-116
Sirt1
Tubulin
1 2 3 4 5
1 2 3 4 5
HEK293T
C2C12 myotubes
B
WT
3A
FLAG-PGC-1α
RSV
kDa
FLAG IP
Input
Acetyl lysine
PGC-1α
PGC-1α
GFP-Sirt1
Tubulin
1 2 3 4 5 6
C
DMSO
RSV
Acetylated PGC-1 α/PGC-1α
NS
***
NS
WT
3A
3D
D
shCtrl DMSO
shCtrl RSV
shSirt1 DMSO
shSirt1 RSV
shLKB1 DMSO
shLKB1 RSV
mRNA levels
(relative expression)
p=0.0517
PGC-1α
NRF-1
NRF-2
NDUFS8
SDHb
Uqcrc1
COX5b
ATP5a1
E
mRNA levels
(relative expression)
****
***
****
****
Sirt1
LKB1
F
shCtrl
shSirt1
rWT
r3A
r3D
kDa
RSV
FLAG-Sirt1
Endo Sirt1
Tubulin
1 2 3 4 5 6 7 8 9 10
G
shCtrl
shSirt1
shLKB1
mtDNA
(relative amount)
NS
NS
DMSO
RSV
H
shCtrl
shSirt1
shLKB1
kDa
RSV
LKB1
Sirt1
Tubulin
1 2 3 4 5 6
I
shSirt1
WT rescue
3A rescue
3D rescue
kDa
RSV
FLAG-Sirt1
Endo Sirt1
Tubulin
1 2 3 4 5 6 7
J
TMRM
454
709
738
unstim.
rWT
rWT+RSV
368
1895
1913
r3A
r3A+RSV
r3D

Figure S5. LKB1-mediated Sirt1 activation promotes mitochondrial function. *A*, Knockdown efficiency of lentivirus-based Sirt1 shRNAs analyzed by Western blotting in HEK293T and C2C12 myotubes. The h1-h4, four shRNAs for HEK293T cells. The m1-m4, four shRNAs for C2C12 myotubes. The selected Sirt1 shRNA used in HEK293T was shSirt1-h1 and shSirt1-m1 was used in C2C12 myotubes. *B*, Deacetylation of PGC-1α by Sirt1 in resveratrol-treated Sirt1-depleted HEK293T cells expressing wild-type Sirt1 or 3A mutant. The experimental replicate of Fig. 5B. *C*, Quantification of acetylated PGC-1α as in Fig. 5B. Data represent mean ± SD. The experiment was repeated three times independently. ***p=0.0002. NS (not significant) indicates p>0.05. Unpaired two-tailed *t*-test for DMSO treatment and resveratrol treatment pair in each group. *D*, The mRNAs were analyzed by means of quantitative PCR in resveratrol-treated LKB1- or Sirt1-depelted C2C12 cells. Data represents mean ± SD. The experiment was repeated three times independently. Statistical significance was determined by Tukey's multiple comparisons test. The p values (shCtrl DMSO, shCtrl RSV) from left to right were 0.0797, 0.0014, 0.0012, 0.0032, 0.0192, 0.0047, 0.0143, 0.0252. The p values (shCtrl RSV, shSirt1 RSV) from left to right were 0.0069, 0.0068, 0.0044, 0.0017, 0.0155, 0.0039, 0.0241, 0.0164. The p values (shCtrl RSV, shLKB1 RSV) from left to right were 0.0093, 0.0089, 0.0009, 0.0025, 0.0441, 0.0056, 0.0517, 0.011. *E*, Sirt1 and LKB1 mRNA were analyzed by means of quantitative PCR in resveratrol-treated Sirt1- or LKB1-depelted C2C12 cells, related to Fig. S5D. Relative expression values were normalized to untreated cells. Data represents mean ± SD. The experiment was repeated three times independently. Statistical significance was determined by Tukey's multiple comparisons test. ***p=0.0002, ****p<0.0001, RSV, resveratrol. *F*, Knockdown efficiency of lentivirus-based Sirt1 shRNA and lentivirus-based expression of Sirt1 in Sirt1-depleted C2C12 myotubes analyzed by Western blotting, related to Fig. 5C and Fig. 5D. Endo Sirt1, endogenous Sirt1. *G*, Mitochondrial content analyzed by means of quantitative PCR in C2C12 cells. Data represents mean ± SD. The experiment was repeated three times independently. Statistical significance was determined by unpaired two-tailed t-test. NS (not significant) indicates p>0.05. *p (shCtrl, shCtrl RSV) =0.0144. p (shSirt1, shSirt1 RSV) =0.3232. p (shLKB1, shLKB1 RSV) =0.2734. *H*, Knockdown efficiency of lentivirus-based LKB1 shRNA and Sirt1 shRNA analyzed by Western blotting in C2C12 myotubes, related to Fig. S5G. *I*, In C2C12 cells endogenous Sirt1 was knocked down, then shRNA-resistant wild-type Sirt1, Sirt1 3A, and Sirt1 3D were expressed. The expression levels of Sirt1 were examined by Western Blot. *J*, The corresponding distribution of TMRM fluorescent dye in Fig. 5G. Scale bar, 10 μm.
